# Structure–Activity Relationships of Pyrrolyl-Containing Diketo Acid and Non-Diketo Acid Derivatives as Inhibitors of SARS-CoV-2 nsp13-Associated Activities

**DOI:** 10.3390/molecules31132376

**Published:** 2026-07-06

**Authors:** Elisa Patacchini, Francesco Saccoliti, Roberta Emmolo, Valentina Noemi Madia, Emanuele Cara, Aurora Albano, Angela Corona, Enzo Tramontano, Roberto Di Santo, Roberta Costi

**Affiliations:** 1 Dipartimento di Chimica e Tecnologie del Farmaco, Istituto Pasteur-Fondazione Cenci Bolognetti, “Sapienza” Università di Roma, p.le Aldo Moro 5, 00185 Rome, Italy; elisa.patacchini@uniroma1.it (E.P.); emanuele.cara@uniroma1.it (E.C.); aurora.albano@uniroma1.it (A.A.); roberto.disanto@uniroma1.it (R.D.S.); roberta.costi@uniroma1.it (R.C.); 2 Department of Life Sciences, Health and Health Professions, Link Campus University, Via del Casale di San Pio V 44, 00165 Rome, Italy; 3 Laboratorio di Virologia Molecolare, Dipartimento di Scienze della Vita e dell’Ambiente Sezione Biomedica, Università di Cagliari, Cittadella Universitaria di Monserrato, SS554, 09042 Monserrato, Italy; roberta.emmolo@unica.it (R.E.); angela.corona@unica.it (A.C.); tramon@unica.it (E.T.); 4 Department of Science, Università degli Studi Roma Tre, Viale Guglielmo Marconi 446, 00146 Rome, Italy; valentinanoemi.madia@uniroma3.it

**Keywords:** SARS-CoV-2 inhibition, nsp13, unwinding inhibition, ATPase inhibition, structure–activity relationship, diketo acid, drug discovery

## Abstract

The SARS-CoV-2 pandemic has posed a tremendous burden globally, highlighting the urgent need for new effective antivirals that are possibly useful against future emerging Coronaviruses (hCoVs). In this context, major efforts were focused on the inhibition of highly conserved and essential targets playing a pivotal role in viral replication. Among them, SARS-CoV-2 nsp13 stands out, being the most conserved enzyme within hCoVs. Following our previous reports describing the identification of indole-based diketo acid (DKA) derivatives as SARS-CoV-2 nsp13 inhibitors endowed with antiviral activity, we applied a scaffold hopping strategy to identify new nsp13 inhibitors. Therefore, we investigated a series of 4-phenyl pyrrolyl DKAs and their structural analogs characterized by molecular simplification or DKA isosteric replacement. The derivatives showed potency against both nsp13-associated activities exhibiting measurable IC_50_s in the low micromolar/submicromolar range, highlighting a promising dual inhibitory profile accordingly. Structure–activity relationship (SAR) studies were performed, highlighting the main structural features increasing the activity of the different compound classes. Interestingly, SAR trends were confirmed in the presence of the BSA/TCEP system despite variations in potency. To shed light on the interaction of the best acting compounds **13b**, **15a**, and **17d**, docking studies were performed, suggesting a putative binding mode in agreement with our previous findings.

## 1. Introduction

The emergence of the severe acute respiratory syndrome coronavirus 2 (SARS-CoV-2) in late 2019 initiated an unprecedented global health crisis [1,2]. While the acute phase of the pandemic has transitioned toward an endemic state, the virus continues to pose a significant challenge due to the persistence of long-term Coronavirus Disease 2019 (COVID-19) [3,4] cases and the continuous emergence of new infections [5,6,7]. A primary driver of this persistence is the rapid evolution of the viral spike protein, which facilitates immune evasion and reduces the long-term efficacy of current vaccines. Consequently, the development of direct-acting antivirals remains a critical necessity to complement vaccination strategies and prepare for potential future outbreaks [8].

To date, clinical intervention has largely focused on two key viral targets: the main protease (M^pro^) [9,10] and the RNA-dependent RNA polymerase (RdRp) [11,12]. Although drugs like Remdesivir, Molnupiravir, and Nirmatrelvir have been approved by the US Food and Drug Administration (FDA), they are not without limitations, including suboptimal pharmacokinetics, potential toxicity, and the emergence of drug-resistant viral variants [13,14,15]. Mutations in M^pro^, for instance, have already been documented to confer resistance to Nirmatrelvir, underscoring the urgent need for next-generation antivirals targeting alternative, highly conserved viral proteins [16,17].

In this context, the non-structural protein 13 (nsp13) has emerged as an exceptionally attractive therapeutic target [18,19]. Nsp13 is a superfamily 1B (SF1B) helicase that plays a pivotal role in the viral replication–transcription complex by utilizing ATP hydrolysis to unwind double-stranded RNA or DNA-RNA hybrids in a 5′ to 3′ direction. Structurally, it comprises two canonical RecA ATPase domains (RecA1 and RecA2) along with three unique domains: a zinc-binding domain (ZBD), a stalk domain (SD), and a 1B domain [20,21].

A defining feature of nsp13 is its remarkable degree of sequence conservation across the *Coronaviridae* family [22,23,24]. Notably, there is a 99.8% sequence identity between the nsp13 of SARS-CoV and of SARS-CoV-2, with only a single amino acid difference (I570 vs. V570) across the entire 601-residue protein [25]. Because this enzyme is so highly conserved among diverse coronaviruses, it offers an outstanding opportunity for the development of broad-spectrum inhibitors. Such agents would likely maintain efficacy across current and future SARS-CoV-2 variants and could potentially provide a first line of defense against future zoonotic coronavirus outbreaks. This therapeutic potential is far from coincidental, as viral helicases are already well-validated targets in drug discovery. The clinical and preclinical success of targeting these enzymes is demonstrated by compounds such as Amenamevir, Pritelivir, and Ebselen, which successfully inhibit helicases from herpes simplex virus (HSV) and hepatitis C virus (HCV) [26,27,28]. Furthermore, recent studies have highlighted helicases from polyomaviruses, Zika virus, and MERS-CoV as highly promising antiviral targets, strongly reinforcing the feasibility and validity of targeting nsp13 [29,30,31].

Recent efforts have identified several small-molecule inhibitors of nsp13, including inhibitors of both natural and synthetic origin, ranging from computationally predicted binders to molecules validated *in vitro* [32,33].

Natural compounds have been widely known as source of compounds active against RNA viruses, such as Influenza, HIV, Dengue, Zika, SARS-CoV-2, etc. [34,35,36].

**Figure 1:**
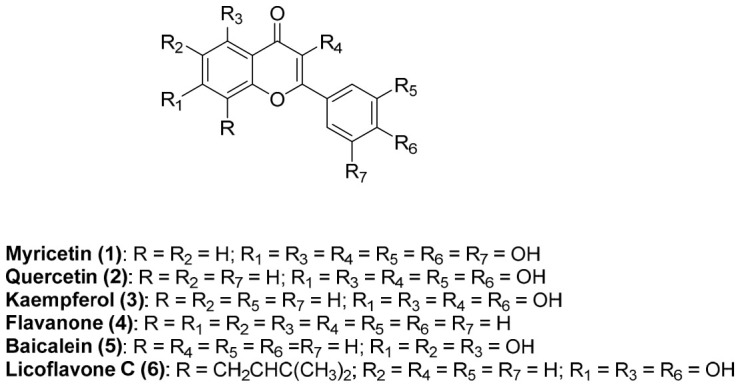
Structures of natural compounds as SARS-CoV-2 nsp13 inhibitors **1**–**6**.

Among them, flavonoids that possess the 3,5-dihydroxychromone motif, were tested against SARS-CoV-2 nsp13 given their activity against SARS-CoV-1 nsp13. Some of them showed low-micromolar inhibition of unwinding activity, such as: Myricetin (**1**, IC_50_ = 0.41 ± 0.11 μM), Quercetin (**2**, IC_50_ = 0.53 ± 0.13 μM), Kaempferol (**3**, IC_50_ = 0.76 ± 0.16 μM), Flavanone (**4**, IC_50_ = 0.52 ± 0.24 μM), Baicalein (**5**, IC_50_ = 2.90 ± 1.0 μM), and Licoflavone C (**6**, IC_50_ = 1.34 ± 0.31 μM) (Figure 1), which also weakly inhibited ATPase activity (IC_50_ = 24.6 ± 3.8 μM). Licoflavone C acts as a non-competitive inhibitor of both nsp13-associated activities with respect to nucleic acid and ATP. However, none inhibited SARS-CoV-2 replication. Moreover, to exclude non-specific aggregation effects, the active compounds were tested against both SARS-CoV-2 nsp13 activities in the presence of bovine serum albumin (BSA) and tris-carboxyethyl phosphine (TCEP). Under these conditions, their inhibitory potency generally decreased, in some cases by up to 50-fold [37].

**Figure 2:**
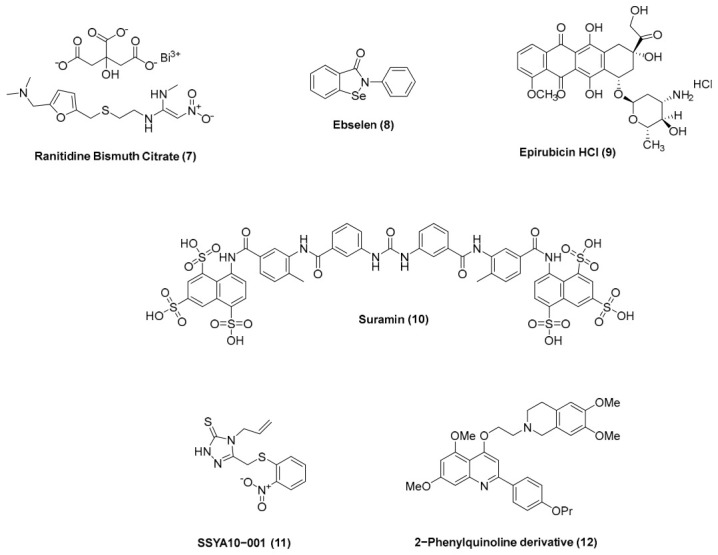
Structures of synthetic compounds as SARS-CoV-2 nsp13 inhibitors **7**–**12**.

Among synthetic compounds, early reports described repurposing FDA-approved compounds while more recent molecules, previously reported against SARS-CoV-1 nsp13, were also described as active against that of SARS-CoV-2. A notable example is Ranitidine bismuth citrate (RBC, **7**, Figure 2), an FDA-approved antiulcer drug, that inhibits both ATPase and the unwinding activities of SARS-CoV-2 nsp13 (IC_50_s of 0.69 ± 0.12 μM and 0.74 ± 0.13 μM, respectively), also demonstrating efficacy in blocking SARS-CoV-2 replication. Kinetic analyses indicated that RBC acts via a competitive inhibition mechanism [38,39]. Another example is Ebselen (**8**, Figure 2), a synthetic organoselenium compound currently in Phase III clinical trials for the treatment of Meniere’s disease. It inhibits SARS-CoV-2 nsp13 ATPase activity with an IC_50_ of 291.9 nM and achieved 50% inhibition of SARS-CoV-2 infected Vero E6 cells at 25 μM [40]. Moreover, four FDA-approved anthracyclines commonly used in cancer chemotherapy were found to inhibit the unwinding activity of SARS-CoV-2 nsp13, with the best acting being Epirubicin HCl (**9**, Figure 2) with an IC_50_ of 0.31 ± 0.03 μM [41].

Among synthetic compounds, promising results have been obtained with Suramin (**10**, Figure 2), which emerged from an HTS that included 0.02% Tween-20 in the assay, a non-ionic detergent that reduces colloid formation. It reported similar IC_50_ values vs. unwinding both in the absence (IC_50_ = 0.94 μM) and in the presence of Tween-20 (IC_50_ = 1.1 μM). It has been hypothesized that Suramin may interact with a positively charged pocket within the enzyme, suggesting that Suramin could target the nucleic acid-binding site of SARS-CoV-2 nsp13 [32,33,42]. One of the most studied inhibitors is the 1,2,4-triazole SSYA10-001 (**11**, Figure 2), originally identified as a SARS-CoV-1 nsp13 unwinding non-competitive inhibitor, without affecting ATPase activity or nucleic acid binding. It showed low cytotoxicity (CC_50_ > 250 μM) and pan-CoV activity, inhibiting SARS-CoV, MERS-CoV, and MHV with EC_50_ values of ~7, 25, and 12 μM, respectively [43]. A subsequent study used SSYA10-001 as a positive control in nsp13 inhibitor screens, with IC_50_ values ranging from 0.05 to 1.73 μM depending on assay conditions, like the use of BSA and TCEP to prevent non-specific aggregation [37]. More recent studies reported the inhibition of both nsp13 unwinding (IC_50_ = 1.8 μM) and ATPase (IC_50_ = 3.78 μM). Time-of-addition experiments indicated that it binds outside RNA and ATP sites, probably in an allosteric pocket within the RecA2 domain [44]. Moreover, a series of 2-phenylquinolines were recently found to inhibit SARS-CoV-2 nsp13 unwinding without affecting ATPase, with the best acting one (**12**, Figure 2), reporting IC_50_s of 0.42 ± 0.23 μM and >30 μM vs. unwinding and ATPase, respectively. It also showed antiviral activities: EC_50_s of 8.8 ± 5.6 μM, 2.9 ± 0.05 μM, and 1.5 ± 0.1 μM against SARS-CoV-2, HCoV-229E, and HCoV-OC43, respectively, even if endowed with some cytotoxicity (CC_50_ of 26.7 ± 4.1 μM in HEL 299 cells) [45].

Although the development of SARS-CoV-2 nsp13 inhibitors has advanced considerably, critical knowledge gaps persist. Notably, a comprehensive evaluation of dual inhibitory effects on both the ATPase and unwinding functionalities is lacking for numerous documented compounds. Because many potent or weak inhibitors were evaluated against only a single activity, a thorough understanding of their binding sites and molecular interactions remains constrained. Moreover, most of the reported compounds suffer from physicochemical liabilities. This is particularly important for compounds identified as potentially “promiscuous” or PAINS, like Ebselen and Suramin (that could have non-specific colloidal effects, even though Suramin is not listed in any PAINS database) [46,47], and natural flavonoids like Myricetin [48]. For these compounds, the observed inhibition often relies on non-specific interactions, including the covalent modification of reactive cysteines or structural disruption of the enzyme’s zinc-finger motifs, rather than competitive engagement at the active site. Poor physicochemical profiles typically explain why many such hits fail in biological systems. A classic example is found in polyphenol-based inhibitors, which generally lack antiviral efficacy in cellular models due to poor metabolic stability and low membrane penetration. Moreover, data suggest a high prevalence of false positives among reported inhibitors, where the measured activity is merely a byproduct of colloidal aggregation in the assay solution, leading to non-specific interactions with the enzyme instead of true, targeted inhibition [32,33,42]. Another study proposed that the inhibition observed for certain compounds, such as anthracyclines, may result from intercalation into the double-stranded nucleic acid substrate rather than direct inhibition of nsp13 helicase activity [49].

As a result of these challenges, identifying promising nsp13 inhibitors remains a complex task.

**Figure 3:**
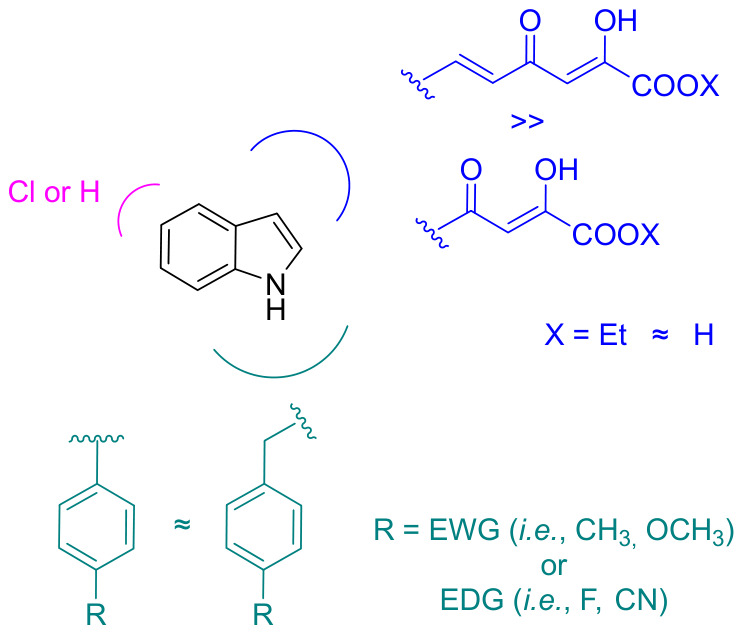
Chemical features of previously reported indolyl DKAs modulating their anti-nsp13 properties.

Recently, we described the first report of small molecules, in particular a set of indolyl diketo acid (DKA) derivatives, acting as potent SARS-CoV-2 nsp13-associated activities inhibitors, together with submicromolar broad-spectrum activity against SARS-CoV-2, MERS-CoV, and HCoV-229E [50]. Building upon these results, we more recently reported two sets of indolyl DKAs endowed with potency in the low micromolar/submicromolar range against unwinding, ATPase, and anti-SARS-CoV-2 effects (Figure 3) [51,52]. Thanks to the data obtained we were able to rationalize structure–activity relationships. We found that on the indolyl scaffold, the introduction at the 3-position of a 4- or 6-carbons DKA/diketo ester branch led to many derivatives endowed with a promising inhibitory profile on the enzymatic target. In particular, the presence of the diketo ester side chain generally improves the inhibition of the unwinding activity, ATPase activity, or both. Specifically, the presence of a diketohexenoic branch is amenable for higher potencies. In addition, the introduction of variously substituted benzyl or aryl rings in position 1 of the indole core confers good inhibitory profiles against unwinding, ATPase, or both, even though slightly better results in inhibiting unwinding were observed. It can be also noticed that various groups endowed with electron-withdrawing/donating or steric properties can be introduced in the para position of the benzyl or aryl ring.

It is also worthy of note that the therapeutic potential of DKA derivatives as antiviral agents has been widely demonstrated in antiviral drug discovery. In particular, our works extensively highlighted the ability of DKAs to inhibit HIV-1 integrase (IN) and/or ribonuclease H (RNase H) and exert antiviral effects [53,54].

Based on these promising findings and leveraging our longstanding expertise on DKAs [55], we aimed to explore different scaffolds for the development of new promising nsp13 inhibitors retaining effective inhibitory potencies without exerting non-specific colloidal effects or physicochemical liabilities.

**Figure 4:**
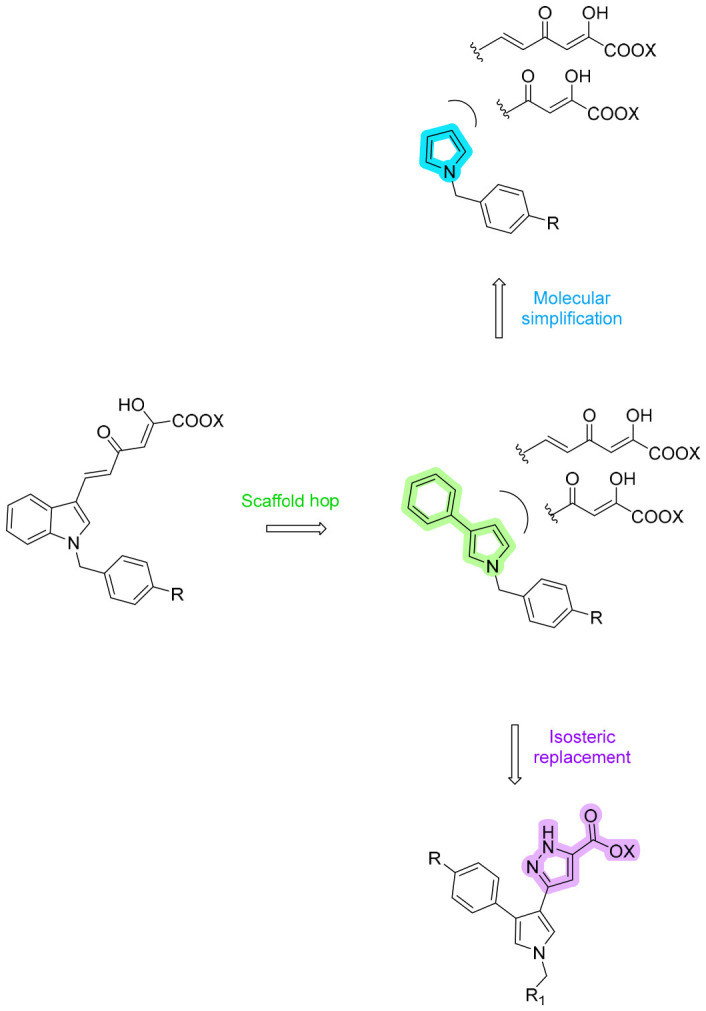
Design of the newly identified pyrrolyl DKA and non-DKA derivatives.

Therefore, we decided to apply a scaffold hopping strategy to our previously reported indolyl DKAs via a ring opening approach. To this end, we investigated a set of 4-phenyl pyrrole DKA derivatives and their respective molecular simplification analogs previously reported as inhibitors of IN and/or ribonuclease H with anti-HIV-1 effects [53,54], all bearing a *N*-substituted benzyl ring similar to our recently reported *N*-benzyl indolyl DKA derivatives. Additionally, we applied an isosteric approach by evaluating a set of pyrrolyl–pyrazole derivatives, previously described to inhibit RNase H and disrupt HIV-1 replication [56], as isosteres of DKA derivatives (Figure 4). It has been reported, in fact, that the isosteric replacement of the DKA chain with a pyrazole carboxylic acid moiety improves chemical stability [57].

**Figure 5:**
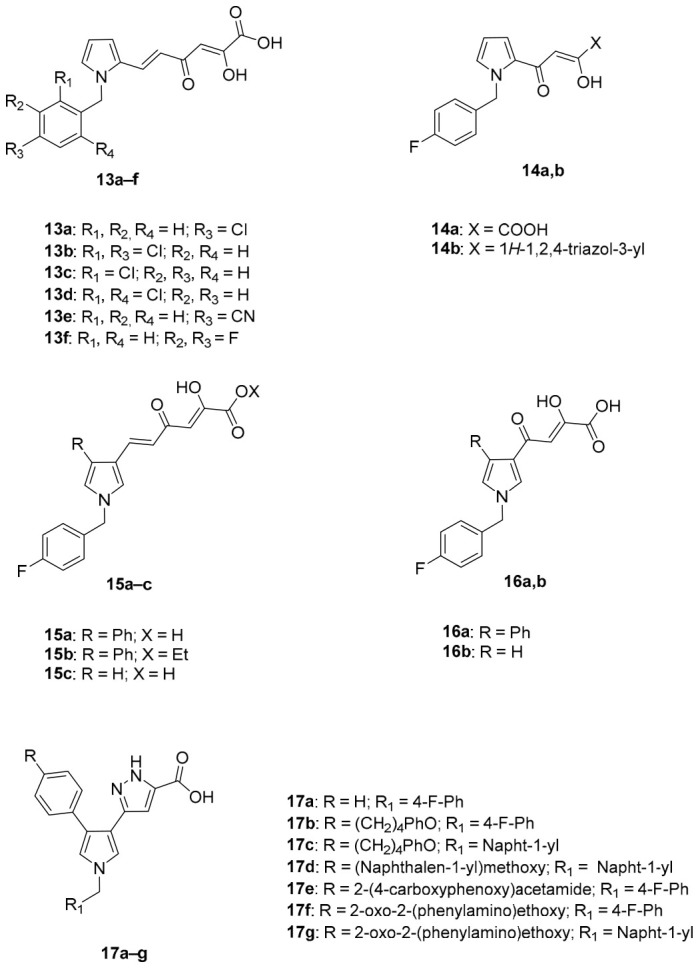
Structures of pyrrolyl DKA and non-DKA derivatives **13a**–**f**, **14a**,**b**, **15a**–**c**, **16a**,**b**, and **17a**–**g**.

In the light of the above, the 20 selected compounds (Figure 5) can be clustered in five groups: (i) pyrrolyl derivatives bearing a diketohexenoic branch in position 2 of the central core and representative substituents on the *N*-benzyl moiety, referred to herein as 2-DKEs (**13a**–**f**); (ii) the 2-diketobutanoic (2-DKB) pyrrole **14a** and its triazolyl analog **14b**; (iii) pyrrolyl derivatives featuring a 3-diketohexenoic side chain, referred to as 3-DKEs (**15a**–**c**); (iv) pyrrolyl compounds endowed with a 3-diketobutanoic moiety, referred to herein as 3-DKB (**16a**,**b**); and (v) a series of isosteric pyrrolyl–pyrazole carboxylic acid derivatives (**17a**–**g**) characterized by diverse *N*-arylalkyl groups and substituents with varying degrees of freedom or steric hindrance.

We evaluated the biological effects of our in-house pyrrolyl-containing DKA and non-DKA derivatives on SARS-CoV-2 nsp13-associated unwinding and ATPase activities. To rule out non-specific inhibition due to aggregation, the most promising compounds were tested under alternative assay conditions. Based on these findings, we preliminarily investigated the structure–activity relationships (SARs) within the various compound series.

## 2. Results and Discussion

### 2.1. Chemistry

Derivatives **13a**–**f**, **14a**,**b**, **15a**–**c**, **16a**,**b**, and **17a**–**g** have been obtained as reported in the literature [53,54,56].

### 2.2. In Vitro Screening for nsp13 Inhibitory Activity

Compounds **13a**–**f**, **14a**,**b**, **15a**–**c**, **16a**,**b**, and **17a**–**g** were tested *in vitro* on both the SARS-CoV-2 nsp13-associated unwinding and ATPase activities using two distinct assay conditions (Table 1, Figures S1 and S2).

Compound SSYA10-001, reported to inhibit SARS-CoV nsp13 [58,59], was used as a positive control, showing an IC_50_ value of 46 nM on SARS-CoV-2 nsp13-associated unwinding activity. Derivatives were also tested on the SARS-CoV-2 nsp13-associated ATPase activity using licoflavone C as a positive control, displaying an IC_50_ value of 24.6 µM on SARS-CoV-2 nsp13-associated ATPase activity.

Based on the primary assay results, pyrrolyl derivatives demonstrated prominent inhibitory potency against nsp13-associated unwinding activity. Indeed, even though no compound outperformed the reference inhibitor SSYA10–001, most derivatives highlighted IC_50_ values lower than 20 μM, with some achieving submicromolar potency.

In contrast, nearly all compounds showed IC_50_s no lower than 30 μM against ATPase activity, proving less active than the ATPase inhibitor licoflavone C. Consequently, their potency was estimated based on percentage of residual enzymatic activity (%residual). Notably, derivative **15a** was the sole exception to this trend, exhibiting inhibitory potency at low micromolar concentrations and proving to be seven times more active than licoflavone C.

To exclude the possibility of inhibition by non-specific aggregators, the best-acting compounds, including both derivatives displaying IC_50_ < 1 μM vs. nsp13-associated unwinding activity and those producing enzymatic residual activity lower than 70% vs. nsp13-associated activity, were further evaluated in different assay conditions (*i.e*., in the presence of 10 μg/mL of BSA and 180 μM TCEP) (Table 1) (Figures S3 and S4). Despite variations in inhibitory potency, the compounds consistently inhibited both nsp13 functions at low micromolar concentrations, highlighting a relevant dual inhibitor profile.

Because of the different ranges of activities across the different assay conditions, the SARs of the pyrrolyl derivatives **13a**–**f**, **14a**,**b**, **15a**–**c**, **16a**,**b**, and **17a**–**g** are discussed separately for unwinding and ATPase activities.

#### 2.2.1. Inhibition of Unwinding

Based on the results obtained in the primary assay, derivatives **13a**–**f**, **14a**,**b**, **15a**–**c**, **16a**,**b**, and **17a**–**g** displayed very promising results on np13-associated unwinding activity, with IC_50_ values ranging from 0.28 ± 0.19 μM to 21.01 ± 6.4 μM (Table 1, Figure S1). While 5 out of the 20 tested compounds showed double-digit micromolar IC_50_s, 8 derivatives exhibited activity at single digit micromolar concentrations, with IC_50_ values below 5 μM (1.42 ± 0.25 µM < IC_50_ < 3.70 ± 1.9 µM). More notably, seven compounds proved to be active at submicromolar concentrations, with IC_50_ values ranging from 0.28 ± 0.19 μM to 0.89 ± 0.12 μM.

Prominent inhibitory activities were highlighted by DKE derivatives from series **13**. In particular, three of the six tested DKEs proved to be active in the submicromolar range, two showed IC_50_s lower than 5 µM, and only one showed an IC_50_ > 10 µM.

To rigorously validate the observed SAR trends, a one-way ANOVA followed by Tukey’s multiple comparisons tests were performed (Table S5).

Preliminary, SARs obtained for the 2-DKE series indicated that the insertion of chlorine atoms on the benzyl ring enhances inhibitory potency. In particular, the 4-Cl derivative **13a** showed high inhibitory activity (IC_50_ = 0.28 ± 0.19 μM), displaying the lowest IC_50_ value across the tested series. Introducing a second chlorine atom at position 2 of the benzyl ring yielded derivative **13b**, which displayed comparable activity to **13a** (IC_50_ = 0.29 ± 0.19 μM). On the other hand, shifting the chlorine substituents to other positions (*i.e*., the 2- and 2,5-positions of the benzyl ring in derivatives **13c**,**d**) increased the IC_50_ values, though these differences were not statistically significant compared to **13a** (*p*-value ≥ 0.9994). Replacing the 4-chloro atom with a 4-cyano group in analog **13e** also resulted in a non-significant increase in IC_50_, suggesting that the cyano group preserves inhibitory activity. In contrast, the insertion of fluorine atoms as alternative halogen substituents on the benzyl moiety significantly reduced inhibitory potency. This was highlighted by the 3,4-difluorosubstituted derivative **13f**, which showed an IC_50_ of 11.70 ± 1.0 μM, making it significantly less potent (approximately 42-fold less) than **13a** (*p*-value < 0.0001). Overall, these preliminary findings indicate that the nature and position of substituents on the *N*-benzyl moiety are likely important features impacting the inhibitory potency of the 2-DKE series.

Unlike 2-DKEs, the 2-DKB derivative **14a** and its triazolyl analog **14b** showed lower potency against unwinding, with IC_50_s higher than 10 μM, making them approximately 75-fold less active than the 2-DKE compound **13a**. The significantly reduced potency of derivatives **14a**,**b** relative to the compounds of series **13** suggests that maintaining the DKA in the 2-position while shortening the DKE branch into a DKB moiety results in reduced unwinding inhibition. Even though compound **14b** displayed a lower mean IC_50_ value than **14a**, the difference was not statistically significant (*p*-value = 0.1404), whereas both derivatives turned out to be significantly less potent than the 2-DKE compound **13a** (*p*-value < 0.0001).

The 3-DKE derivatives **15a**–**c** showed low micromolar to high submicromolar IC_50_s, displaying significantly improved potency against unwinding compared to the 2-DKB compounds **14a**,**b** (*p*-value < 0.0001), while no statistically significant differences in potency compared to the 2-DKE compounds **13a**–**e** were observed. The 3-DKE derivative **15c** (IC_50_ = 2.52 ± 1.2 μM) turned out to be nearly eight times more active than its 2-DKB analog **14a** (IC_50_ = 21.01 ± 6.4 μM), suggesting that shifting and elongating the 2-DKB chain into a 3-DKE moiety could be a more effective strategy to inhibit unwinding at low micromolar concentrations. This agrees with our previous findings about indolyl DKAs, where the DKE derivatives demonstrated more potent inhibition than the corresponding DKBs.

Structural modifications on the 3-DKE scaffold through the insertion of a phenyl group at position 4 of the pyrrole ring (**15b**) or the replacement of the terminal carboxyl with an ester group (**15a**) slightly improved the IC_50_ values, even though no statistical difference in their inhibitory potency was detected (*p*-value > 0.9999).

Similar to the 3-DKEs, the 3-DKB derivatives **16a**,**b** exhibited inhibitory activity in the micromolar range. However, unlike in the 3-DKE series, introducing a 4-phenyl group to the 3-DKB scaffold significantly improved the potency. Specifically, the phenyl-substituted derivative **16a** (IC_50_ = 2.40 ± 0.59 μM) showed a 5-fold increase in potency compared to its unsubstituted counterpart **16b** (IC_50_ = 12.90 ± 2.8 μM; *p*-value < 0.0001). This finding indicates that such structural modification significantly enhances the potency of the 3-DKB series, while only displaying marginal effects in the 3-DKEs. Overall, we can state that the scaffold hopping approach could be a promising strategy for the development of nsp13 inhibitors and that molecular simplification could be applied as well, even if it affects the activity to different extents depending on the length of the DKA chain.

Additionally, the 3-DKB derivatives proved superior to both the 2-DKB compound **14a** and its triazolyl analog **14b**. Specifically, compound **16b** (IC_50_ = 12.90 ± 2.8 μM) displayed a statistically significant, nearly two-fold higher potency than its 2-DKB counterpart **14a** (IC_50_ = 21.01 ± 6.4 μM; *p*-value = 0.0005), suggesting that the regiochemistry of the DKB branch on the pyrrole core could be a critical determinant for unwinding inhibition.

To investigate the isosteric replacement of the DKA group with a pyrazole-carboxylic acid moiety, a set of pyrazole derivatives **17a**–**g** with alternative groups in position 4 of the pyrrole core was also assessed. In addition to the direct 4-phenyl non-DKA analog of **16a** (*i.e*., derivative **17a**), we examined a set of oxyphenylpyrrolyl−pyrazole derivatives, **17b**–**g,** featuring substituents with different degrees of freedom or steric hindrance on the 4-oxyphenyl moiety, along with a methylnaphth-1-yl or a *p*-fluorobenzyl ring on the pyrrolyl nitrogen.

The compounds showed promising inhibitory potency on np13-associated unwinding activity, highlighting IC_50_ values ranging from 0.35 ± 0.042 μM to 16.40 ± 3.6 μM. Among the seven tested pyrazoles, only one compound (**17e**) showed activity in the double-digit micromolar range. In contrast, three derivatives (**17a**,**f**,**g**) exhibited IC_50_s below 5 μM, and, most notably, three compounds (**17b**–**d**) highlighted submicromolar potency against unwinding.

Comparing the 4-DKB compound **16a** (IC_50_ = 2.40 ± 0.59 μM) with its pyrazole analog **17a** (IC_50_ = 3.70 ± 1.9 μM) revealed similar potencies (*p*-value > 0.9999). This finding indicated that replacing the DKA moiety with a pyrazole-based isostere preserves the inhibitory activity, preliminarily suggesting this modification as a viable strategy to achieve unwinding inhibition.

Notably, replacing the 4-phenyl group of compound **17a** with *O*-substituted 4-oxyphenyl moieties in derivatives **17b**–**g** generally decreased IC_50_ values against unwinding, although formal statistical analysis revealed that only compound, **17e**, differed significantly from **17a**–**d**,**f**,**g** (*p*-value < 0.0001).

Based on the measured IC_50_ values, it can be preliminarily observed that the introduction of a phenylbutyl group at the 4-oxyphenyl position yielded derivatives **17b**,**c**, which displayed IC_50_s in the submicromolar range. Furthermore, the nature of the arylmethyl group at position 1 of the pyrrole core seemed to influence the overall activity. Indeed, while the insertion of a (naphthalen-1-yl)methyl group in derivative **17c** (IC_50_ = 0.60 ± 0.5 μM) resulted in a nearly 6-fold gain in activity relative to **17a**, apparently greater potency was found for the *p*-fluorobenzyl-substituted compound **17b** (IC_50_ = 0.35 ± 0.042 µM), which proved to be nearly 10-fold more potent than **17a** and twice as active as **17c** (IC_50_ = 0.60 ± 0.5 µM).

In addition, the specific nature of the 4-oxyphenyl substituents seemed to influence inhibitory potency; accordingly, pyrazole derivatives bearing alternative ether appendages exhibited distinct IC_50_ values. In this regard, despite remaining active in the submicromolar range, the *bis*-(methylnapht-1-yl) derivative **17d** (IC_50_ = 0.89 ± 0.12 μM) showed a nearly two-fold lower potency than its phenylbutyl analog **17c** (IC_50_ = 0.60 ± 0.5 μM). Higher IC_50_s were observed for derivatives **17f**,**g** featuring an *N*-phenylacetamide group on the oxyphenyl moiety, resulting in them being approximatively six-fold less active than the phenylbutyl derivative **17b** (**17b**, IC_50_ = 0.35 ± 0.042 μM; **17f**, IC_50_ = 2.13 ± 1.14 μM; **17g**, IC_50_ = 2.10 ± 0.86 μM). On the other hand, despite carrying different *N*-arylalkyl substituents, compounds **17f**,**g** displayed nearly identical IC_50_ values, suggesting that variations at the 1-position play only a marginal role in the activity of this scaffold.

In contrast, the insertion of an additional carboxyl group in position 4 of the *N*-phenylacetamide moiety had a detrimental effect on inhibitory potency. Indeed, derivative **17e** (IC_50_ = 16.40 ± 3.6 μM) showed a statistically significant reduction in activity compared to all the other derivatives in this series (*p*-value < 0.0001), including a four-fold lower activity level than derivative **17a** (IC_50_ = 3.70 ± 1.9 μM) and an eight-fold reduction in potency compared to **17f** (IC_50_ = 2.13 ± 1.14 μM), indicating that a carboxyl group in this position negatively impacts the inhibitory profile.

#### 2.2.2. Inhibition of ATPase

Compounds **13a**–**f**, **14a**,**b**, **15a**–**c**, **16a**,**b**, and **17a**–**g** were also assessed against nsp13-associated ATPase activity in the primary assay (Table 1). Unlike their effects on unwinding, the compounds showed inhibition of ATPase at concentrations of 30 µM or higher (IC_50_ ≥ 30 µM), with %residual ranging from 51% to 96%, while derivative **17a** was inactive (%residual = 100%). A notable exception to this activity trend was the 3-DKE derivative **15a**, which exhibited inhibitory potency within the low micromolar range (IC_50_ = 3.39 µM). Based on this, the compound emerged as the most active ATPase inhibitor of the series, outperforming the reference inhibitor licoflavone C by nearly 7-fold.

Based on %residual, varying inhibitory potencies were observed across the chemical classes allowing for a preliminary discussion of the SARs.

Consistent with the unwinding results, the inhibition of ATPase by the 2-DKEs proved to be affected by both the nature and pattern of substitution of the *N*-benzyl moiety in 2-DKEs. While the overall inhibitory trends for ATPase and unwinding appeared generally aligned, a few key differences emerged.

Notably, the 2,4-dichloro derivative **13b** and the 4-chloro derivative **13a** exhibited the highest descriptive potencies of the series (**13a**: %residual = 58%; **13b**: %residual = 51%), while the 3,4-difluoro compound **13f** showed a lower potency (%residual = 95%). These findings mirror the results of the unwinding assays, suggesting that a 4-Cl substituent may be a beneficial structural feature for the inhibition of nsp13-associated activities.

While 2-DKB **14a** and its triazolyl analog **14b** exhibited low potencies (%residual = 96%), a more interesting profile was highlighted for the 3-DKEs **15a**–**c**. In line with the unwinding results, a comparison between the potencies of the 3-DKEs indicated that the 4-substituent may be relevant for inhibitory activity, with the 4-phenyl substituted derivatives **15a**,**b** being more active than derivative **15c** which was lacking such group. Specifically, while derivative **15c** resulted in a %residual of 86%, the 4-phenyl-substituted analog **15b** yielded a higher apparent inhibition of ATPase (%residual = 51%), showing comparable potency to the 2-DKE derivative **13b**. An even greater activity was highlighted for the ester **15a**, which outperformed all the tested compounds as the only derivative with potency against ATPase in the low-micromolar range (IC_50_ = 3.39 ± 0.16 µM).

Conversely, the 3-DKB compounds **16a**,**b** exhibited weaker enzyme inhibition (%residual = 81–88%), with the 4-unsubstituted derivative **16b** being slightly more potent than the 4-phenyl analog **16a**. This preliminary finding indicated that the 4-phenyl substituent might influence the potency of 3-DKEs and 3-DKBs differently, while also highlighting an activity trend for ATPase inhibition that is apparently opposite to that observed for unwinding.

A more interesting profile was observed for the pyrazole derivatives **17a**–**g**, which displayed a broader range of potencies (%residual = 59–100%). While compound **17a** was inactive (%residual = 100%), derivatives **17b**–**g** displayed higher apparent inhibitory potencies against ATPase, with the activity trend seemingly influenced by the substituents on both the 4- and 1-positions. Indeed, the insertion of a phenylbutyl moiety on the 4-oxyphenyl group in derivatives **17b**,**c** resulted in enhanced inhibitory potencies relative to **17a**, with the 4-fluorobenzyl derivative **17b** proving slightly more active (%residual = 63%) than its methylnapht-1-yl analog **17c** (%residual = 68%), while showing very similar potency to the *bis*-(methylnapht-1-yl) analog **17d** (%residual = 61%). This finding likely suggests a differential impact on nsp13-associated activities, where such a substitution pattern may more significantly affect ATPase than unwinding.

The insertion of *N*-phenylacetamide or *N*-phenyl-4-carboxyacetamide groups on the 4-oxyphenyl moiety resulted in different apparent inhibitory potencies, which appeared to be dependent on the substituent at position 1 of the pyrrole core. Indeed, while the 4-fluorobenzyl group resulted in limited potency for derivatives **17e**,**f** (%residual = 82–88%), the combination of a phenylacetamide group with a 1-methylnaphthyl moiety yielded the most potent derivative in the pyrazole series, **17g** (%residual = 59), suggesting a potential synergistic effect between these groups on ATPase inhibition.

#### 2.2.3. Inhibition of Unwinding and ATPase in the Presence of BSA and TCEP

Following the primary assays, compounds showing high potency against nsp13-associated activities were tested under different conditions to rule out inhibition by non-specific aggregators. In particular, derivatives **13a**–**c**, **15a**,**b**, and **17b**–**d**,**g**, which displayed either IC_50_ < 1 µM vs. unwinding, low single digit micromolar potency vs. ATPase, or caused a %residual lower than 70% vs. ATPase, were also tested in the presence of 10 μg/mL of BSA and 180 μM TCEP (Table 1).

To rigorously validate the observed SAR trends, a one-way ANOVA followed by Tukey’s multiple comparisons tests were performed (Tables S6 and S7).

Regarding unwinding activity, the selected compounds displayed IC_50_ values within the micromolar range, showing a general decrease in inhibitory potency compared to the primary assays, suggesting that the BSA/TCEP system influences the efficacy of the derivatives. As previously reported for some natural compounds, BSA can affect both compound potency and enzyme catalytic performances, producing variations by altering *k*_cat_ and *K*_m_ values. This indicates a direct effect of BSA on the enzyme, potentially modifying its conformation distributions and thereby affecting small molecule binding to nsp13 [32]. Moreover, BSA can influence assay readouts through multiple, compound-specific mechanisms: reversible or irreversible binding to BSA (sequestration), the stabilization or destabilization of labile compounds, the alteration of compound aggregation equilibria (promoting or inhibiting colloidal aggregation), and modulation of the local microenvironment. TCEP can additionally affect the activity of redox-sensitive compounds by reducing disulfides or altering metal-coordination states. Therefore, the final observed effect can be the result of the sum of these factors, and the relative SAR evaluation has been performed independently for the new conditions.

Notably, this activity trend was also observed for the positive control, SSYA10–001, which exhibited an IC_50_ value of 1.73 μM on SARS-CoV-2 nsp13 unwinding activity under this assay conditions, displaying a 37-fold reduction in potency compared to the value obtained in the absence of BSA/TCEP [32].

Despite the variations in activity, compounds maintained prominent inhibitory potencies under these conditions, with IC_50_ values below 10 µM.

Overall, all the tested compounds were active against unwinding in the same order of magnitude as SSYA10-001. Specifically, five compounds (**13c**, **15a**,**b**, **17b**,**g**) were significantly (approximatively 3- to 5-fold) less potent than the reference compound (**13c**: *p*-value < 0.0001; **15a**: *p*-value = 0.0142; **15b**: *p*-value = 0.05; **17b**: *p*-value < 0.0001; **17g**: *p*-value: = 0.0027). On the other hand, although derivatives **13a** and **13b** exhibited a nearly two-fold increase in IC_50_ values relative to SSYA10-001, these differences were not statistically significant (0.6384 < *p*-value < 0.8845), while compound **17c** demonstrated a comparable potency to SSYA10-001 (*p*-value > 0.9999). Finally, derivative **17d** displayed an approximate 2-fold lower IC_50_ value than SSYA10-001, even though this enhanced activity did not reach statistical significance (*p* = 0.9998).

Overall, the qualitative activity trends and relative SAR patterns detected for the unwinding under such assay conditions remained generally consistent with those observed in the absence of BSA and TCEP.

Regarding the 2-DKEs, derivative **13a** was confirmed as the compound from series **13** with the lowest IC_50_ values against unwinding under both assay conditions. In addition, derivative **13a** (IC_50_ = 3.10 ± 0.97 µM) demonstrated comparable potency to **13b** (IC_50_ = 3.51 ± 0.69 µM), in addition to being nearly three times more active than derivative **13c** (IC_50_ = 9.50 ± 2.8 µM) across both setups. However, in contrast to the results obtained in the absence of BSA/TCEP, the differences in potency between the 4-chloro derivative **13a** and its 2-chloro isomer **13c** proved to be statistically significant (*p*-value < 0.0001). This finding was also confirmed by comparing the IC_50_ value of the 2,4-dichloro-substituted analog **13b** with that of **13c**, suggesting that the insertion of a 4-Cl substituent is a key structural feature for unwinding inhibition.

Similar to the trend highlighted in the absence of BSA/TCEP, the 3-DKE ester **15a** showed a slightly higher IC_50_ (IC_50_ = 5.39 ± 1.01 µM) than the corresponding acid **15b** (IC_50_ = 4.88 ± 0.19 µM), even though their differences in potency were not statistically significant (*p*-value > 0.9999).

In contrast to the results obtained in the primary assays, the pyrazole derivatives showed a more divergent activity profile against unwinding in the presence of BSA/TCEP. Specifically, while the results from the primary screening indicated no significant statistical differences in the inhibitory potencies of **17b**–**d**, preliminarily suggesting higher activity for compound **17b** relative to analogs **17c**,**d**, an opposite activity trend was highlighted in the experiments conducted in the presence of BSA/TCEP. Accordingly, compound **17d** (IC_50_ = 1.15 ± 0.37 µM) displayed a statistically significant 7-fold higher potency than **17b** (IC_50_ = 8.60 ± 0.1 µM; *p*-value < 0.0001). Conversely, **17d** exhibited no significant difference in potency relative to **17c** (IC_50_ = 2.19 ± 0.49 µM, *p*-value = 0.9784) and **13a**,**b** (0.2702 < *p*-value < 0.5189), ultimately emerging as one of the most potent unwinding inhibitors across all the tested series, and preliminarily suggesting that isosteric modification is a useful strategy to inhibit unwinding.

Interestingly, despite displaying a phenylbutyl group at the 4-oxyphenyl moiety as a common structural feature, derivatives **17b**,**c** displayed significantly different potencies, indicating that the specific substituent in position 1 of the pyrrole core is relevant for activity. In particular, the insertion of a naphthalen-1-ylmethyl group led to the more potent compound **17c** (IC_50_ = 2.19 ± 0.49 µM), while its replacement with a 4-fluorobenzyl moiety in derivative **17b** significantly decreased the inhibitory potency by nearly 4-fold (IC_50_ = 8.60 ± 0.1 µM, *p*-value < 0.0001). Interestingly, derivatives **17c**,**d** bearing the *N*-(naphthalen-1-ylmethyl) group in position 1 of the pyrrole core showed significantly higher potencies than **17b** (*p*-value < 0.0001), suggesting that inserting such substituent can enhance the inhibitory activity. Nevertheless, the *N*-(naphthalen-1-ylmethyl) derivatives **17c**,**d**,**g**, featuring alternative ether linkages, displayed different activities, indicating that the specific substituents at the 4-oxyphenyl group can also influence inhibitory potency. In particular, the insertion of a further *N*-(naphthalen-1-ylmethyl) group in such position led to *bis*-(naphthalen-1-ylmethyl))-substituted derivative **17d** (IC_50_ = 1.15 ± 0.37 µM). This compound displayed a similar, non-significantly different potency than **17c** (IC_50_ = 2.19 ± 0.49 µM, *p*-value = 0.9784), indicating that the phenylbutyl and the naphthalen-1-ylmethyl groups likely exert a similar effect on inhibitory potency. Conversely, inserting an *N*-phenylacetamide group at the 4-oxyphenyl moiety proved to reduce unwinding inhibition, as highlighted by derivative **17g** (IC_50_ = 6.03 ± 1.32 µM) which displayed significantly lower activity compared to both **17c** (*p*-value = 0.009) and **17d** (*p*-value = 0.0006).

Interestingly, while adding BSA/TCEP to the assays reduced the inhibitory potency of compounds against unwinding, an oppositive trend was observed for ATPase activity. Indeed, although none of the tested compounds except **15a** showed IC_50_ values below 30 µM in the primary assay, higher potency (IC_50_ < 10 µM) was highlighted for derivatives **13a**,**b**, **15a**,**b**, and **17b**–**d**,**g** in the presence of BSA/TCEP. In contrast, the inhibitory potency of the ATPase inhibitor licoflavone C remained largely unaffected by the presence of BSA/TCEP, yielding an IC_50_ value comparable to the one obtained in the primary assay. Consequently, compounds **13a**,**b**, **15a**,**b,** and **17b**–**d**,**g** significantly outperformed licoflavone C in inhibiting ATPase function, displaying up to a 16-fold increase in potency with IC_50_ values ranging from 1.84 ± 0.33 µM to 21.90 ± 3.0 µM.

Within the 2-DKE series, derivatives **13a**,**b**, which previously produced a %residual of 58% and 51% against ATPase, demonstrated IC_50_ values of 8.00 ± 3.6 µM and 6.90 ± 2.0 µM respectively, proving to be nearly four times as potent as licoflavone C. In contrast, the 2-DKE derivative **13c** highlighted an IC_50_ of 21.90 ± 3.0 µM, which is consistent with its lower potency against ATPase detected in the first assay (%residual = 84%). Similarly, compound **15b**, which caused a %residual of 51% in the primary assay, showed higher potency (IC_50_ = 6.82 ± 1.16 µM) under the new conditions. Interestingly and similarly to what was observed in the absence of BSA/TCEP, the 3-DKE derivative **15a** exhibited the lowest IC_50_ against ATPase across the different series (with an IC_50_ value of 1.84 ± 0.33 µM), resulting in a 16-fold higher activity level than licoflavone C.

Interestingly, regarding the pyrazole derivatives **17b**–**d**,**g**, the general activity trend on ATPase resembled that already discussed for the unwinding assay in the presence of BSA/TCEP. Accordingly, the *bis*-(naphthalen-1-ylmethyl) derivative **17d** exhibited a lower IC_50_ (IC_50_ = 2.33 ± 0.66 µM) than compounds **17b**,**c**,**g** (**17b**, IC_50_ = 5.20 ± 1.3 µM; **17c**, IC_50_ = 3.60 ± 0.34 µM; **17g**, IC_50_ = 4.98 ± 1.42 µM), even though their differences in potency were not statistically significant.

Finally, it is worth noting that compounds across all series exhibited similar potencies against unwinding and ATPase in the presence of BSA/TCEP, highlighting a consistent dual inhibitory profile. Based on the IC_50_ values, and the relative potency differentials, the following observations can be preliminarily extrapolated.

Within the 2-DKE series, derivatives **13a**–**c** showed a less than 3-fold difference in their potency against unwinding and ATPase, with derivative **13b** highlighting a balanced profile (IC_50_ unwinding = 3.51 ± 0.69 µM; IC_50_ ATPase = 6.90 ± 2.0 µM). This trend extended to the 3-DKE series, where acid **15b** appeared to be another interesting dual inhibitor (IC_50_ unwinding = 4.88 ± 0.19 µM; IC_50_ ATPase = 6.82 ± 1.16 µM). An apparently more pronounced dual inhibitory profile was observed in the pyrazole series, with derivatives **17b**–**d**,**g** generally displaying narrower potency differentials than the other chemical classes. In particular, while compound **17d** showed up to a 2-fold difference (IC_50_ unwinding = 1.15 ± 0.37µM; IC_50_ ATPase = 2.33 ± 0.66 µM), derivative **17g** (IC_50_ unwinding = 6.03 ± 1.32 µM; IC_50_ ATPase = 4.98 ± 1.42 µM) highlighted very similar potencies against nsp13-associated activities (1.2-fold differential), emerging as the best dual inhibitor across all the tested series.

Based on the IC_50_ values highlighted in the biochemical assays, the DKA and non-DKA derivatives display similarities with previously reported inhibitors. Indeed, while seemingly less potent than natural-based unwinding inhibitors like Myricetin, Quercetin, Kaempferol, and Flavanone, the compounds showed inhibitory potencies at single-digit micromolar concentrations, resembling Baicalein and licoflavone C, in addition to being significantly more active than the latter against the ATPase activity.

On the other hand, while the compounds appeared less potent than some synthetic nsp13 inhibitors (such as Suramin, Ebselen, and the 2-phenylquinoline **12**), they displayed IC_50_ values within the same order of magnitude of our previously reported indolyl-DKAs and SSYA10-001, as previously discussed. At the same time, unlike other reported inhibitors, which generally display selectivity against either unwinding or ATPase activity, our compounds exhibited similar IC_50_s against both nsp13-associated functions. This represents a distinctive feature that could potentially enhance their utility in drug development. This encouraging activity profile, combined with the higher synthetic accessibility of our derivatives compared to the generally challenging natural compounds, provides a relevant rationale to prompt additional investigations of these scaffolds.

**Figure 6:**
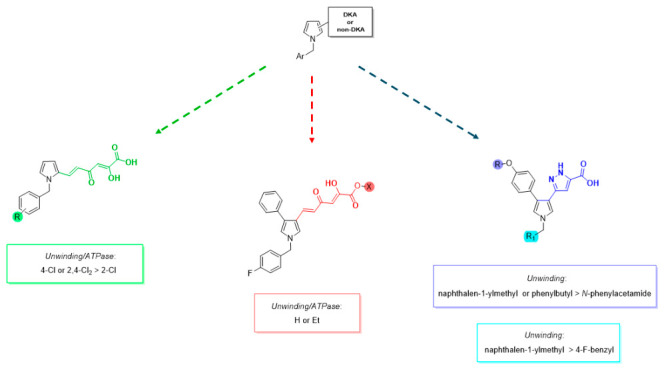
Graphical representation of SAR trends observed in the most promising pyrrolyl DKA and non-DKA derivatives.

Overall, the discussed findings highlighted three series of pyrrolyl-containing DKA and non-DKA derivatives with significant potency against nsp13-associated functions, delineating general activity trends and preliminary SAR insights, as summarized in the Figure 6.

### 2.3. Molecular Docking

**Figure 7:**
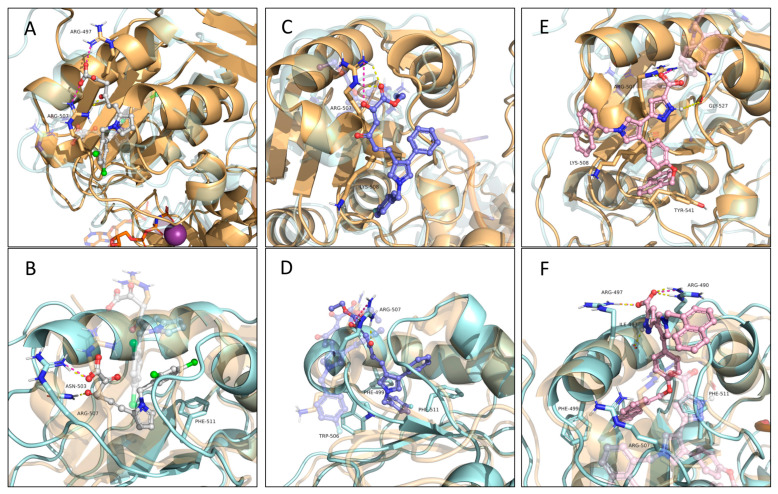
Predicted binding poses for compounds **13b** ((**A**,**B**), cyan sticks), **15a** ((**C**,**D**), blue sticks), and **17d** ((**E**,**F**), pink sticks). The upper panels (**A**,**C**,**E**) illustrate the binding modes obtained using the ATPase inhibition model, while the bottom panels (**B**,**D**,**F**) display the poses generated for the unwinding inhibition model. The proteins are represented as gold and pale cyan cartoons, respectively. For comparative purposes, the alternative binding pose and the corresponding protein conformation are shown in transparency in each panel.

To shed light on the most promising compounds’ binding modes toward SARS-CoV-2 nsp13, molecular docking protocols were developed to closely replicate the experimental conditions of the assays. To evaluate ATPase inhibition, docking was performed using a pre-hydrolysis state model of nsp13 complexed with a non-hydrolyzable ATP analog AMP-PNP and a Mg^2+^ ion. Conversely, unwinding inhibition was investigated using a previously published homology model of SARS-CoV-2 nsp13 [50], derived from the post-hydrolysis structure of SARS-CoV-1 and subsequently refined with the inclusion of ADP and a nucleic acid substrate. Given that previous studies on a similar chemical class [50] have demonstrated competition with the known allosteric inhibitor SSYA10-001, docking simulations were focused on the hypothesized binding site located on the RecA2 domain. Due to a lack of resolved three-dimensional structures complexed with allosteric inhibitors, an Induced Fit Docking (IFD) approach was employed to account for potential conformational adaptations. Regarding ligand preparation, all possible tautomeric forms of the DKA moiety were considered; furthermore, pKa calculations for **15a** confirmed that the enolic form exists predominantly as enolate under physiological conditions. The final binding poses, selected based on the best docking scores and visual inspection, are illustrated in Figure 7.

Compound **13b** (Figure 7A,B) presents two distinct predicted binding modes across the two models employed. In the ATPase inhibition model, the DKA moiety seems to play a primary role in establishing interactions with the basic protein residues Arg-507 and Arg-497. Specifically, it is predicted to form electrostatic interactions, with the chain carbonyl additionally acting as a hydrogen bond acceptor for Arg-507. In the unwinding model, the DKA moiety likely functions as a hydrogen bond acceptor for the side chains of Asn-503 and Arg-507, while also establishing a salt bridge interaction with the latter. Furthermore, the benzylic portion is found in a favorable position to potentially establish π-π stacking interactions with Phe-511.

The Arg-507 residue also appears to be a key residue within the predicted binding poses for derivative **15a** (Figure 7C,D). In the ATPase model, this residue acts as a proposed hydrogen bond donor and interacts electrostatically with the enolate of the DKE chain. The benzylic moiety is positioned in close proximity to Lys-508, suggesting potential cation–π interactions. In the unwinding model, the DKE chain is predicted to interact once again with Arg-507 (both electrostatically via the enolate and by acting as a hydrogen bond acceptor). Moreover, the aromatic benzylic and phenylic functionalities are well accommodated within a lipophilic pocket defined by Phe-499, Trp-506, and Phe-511, where they could easily engage in π-π stacking with these residues.

Finally, Arg-507 is also suggested to be relevant for the binding of the pyrazole derivative **17d** (Figure 7E,F). In the ATPase model, this residue is predicted to form a salt bridge with the carboxylic moiety of the compound. Interestingly, we also observe the formation of a hydrogen bond between the pyrazole NH and the backbone of Gly-527. The methylnaphth-1-yl and naphthalen-1-ylmethoxy moieties are favorably oriented to allow cation–π interactions with Lys-508 or π-π stacking with Tyr-541. In the unwinding model, the pyrazole NH continues to act as a putative hydrogen bond donor, interacting with Ile-493. The carboxylic acid group, conversely, interacts with the basic residues Arg-490 and Arg-497 through ionic bonding and as a hydrogen bond acceptor. Ultimately, the naphthalen-1-ylmethoxy portion is well oriented toward Phe-499, suggesting T-shaped π-π stacking or possible cation–π interactions with Arg-507.

Taken together, the predicted binding modes suggest that the DKA moiety plays a prominent role in maintaining specific molecular interactions with the amino acid residues characterizing this domain. Furthermore, functionalization with aromatic substituents (such as *N*-benzylic or methylnaphth-1-yl groups, or aromatic moieties at position 4 of the pyrrole core) broadens the scope of potential interactions within this pocket. Interestingly, modifying the DKA arm with a pyrazole introduces a novel hydrogen bond donor site. Together, these structural features promote a robust network of H-bonds, electrostatic interactions, and π-stacking, which could potentially stabilize the ligands within the allosteric pocket of the RecA2 domain across both functional states of nsp13.

## 3. Materials and Methods

### 3.1. Chemistry

Derivatives **13a**–**f**, **14a**,**b**, **15a**–**c**, **16a**,**b** and **17a**–**g** were obtained as reported in the literature [53,54,56].

### 3.2. Biological Assays

#### 3.2.1. SARS-CoV-2 nsp13 Expression and Purification

SARS-CoV-2 nsp13 was expressed from a pNIC-ZB vector (addgene 159614) [60] following the procedure described in reference [50]. Briefly, the protein was expressed in E. coli BL21 Rosetta 2 cells in Terrific Broth media induced with 300 µM IPTG and lasted overnight at 18 °C at 200 rpm.

Cell pellets were resuspended and sonicated for 15 min, 10 s on 5 s off, and clarified by centrifugation at 11,000× *g* for 50 min. The supernatant was used for a batch binding of 40 min with 3 mL of Ni-sepharose (Cytiva, Marlborough, MA, USA). Beads were loaded on a gravity flow column and washed with 50 mL lysis buffer, 25 mL wash buffer (50 mM HEPES pH 7.5, 500 mM Nacl, 5% Glycerol, 45 mM imidazole, 0.5 mM TCEP), 10 mL Hi-salt buffer (50 mM HEPES pH 7.5, 1 M NaCl, 5% Glycerol, 0.5 mM TCEP), and again with 10 mL of the wash buffer. The protein was eluted with an elution buffer (50 mM HEPES pH 7.5, 500 mM NaCl, 5% Glycerol, 300 mM imidazole, 0.5 mM TCEP). The eluted fraction was immediately applied to a 5 mL Hi-Trap SP HP column using a syringe. The column was washed with 20 mL elution buffer, and proteins were eluted with 20 mL Hi-salt buffer (20 fractions 0–100% Hi-salt buffer). Protein fractions were pooled and loaded into a Superdex 200 10/300 GL column equilibrated in 50 mM HEPES, 500 mM NaCl, 5% Glycerol, and 0.5 mM TCEP. The elution fractions were analyzed for purity by SDS page. Proteins were stored at −80 °C.

#### 3.2.2. Determination of SARS-CoV-2 nsp13 Unwinding-Associated Activity

The SARS-coV nsp13 unwinding-associated activity was measured as reported [37] in black 384-well plates (PerkinElmer), in 20 μL reaction volume containing 20 mM Tris–HCl pH 7.2, 50 mM NaCl, 2 μM Hel Capture oligo (5′-TGG TGC TCG AAC AGT GAC-3′) from Biomers, 5 mM MgCl_2_, 5% dimethyl sulfoxide (DMSO) or inhibitor, and 2 nM of purified nsp13, with or without 10 µg/mL BSA and 180 µM TCEP. The reaction mixture containing the enzyme was pre-incubated for 10 min with an inhibitor at room temperature (RT). The reaction was started by adding 500 µM ATP and 500 nM annealed DNA substrate (5′-AGT CTT CTC CTG GTG CTC GAA CAG TGA C-Cy3-3′, 5′-BHQ-2-GTC ACT GTT CGA GCA CCA CCT CTT CTG A-3′) from Biomers. After 15 min of incubation at 37 °C, fluorescence products were measured with Victor Nivo (Perkin, Waltham, MA, USA) at 530/580 nm. The experiments were conducted as N greater or equal to 3 biological independent replicates of three technical replicates for each tested condition.

#### 3.2.3. Determination of SARS-CoV-2 nsp13 ATPase-Associated Activity

SARS-CoV-2 nsp13 ATPase-associated activity was measured as reported [37] in a transparent 96-well plate (PerkinElmer, Waltham, MA, USA), in 25 μL reaction volume containing 20 mM Tris–HCl pH 7.2, 50 mM NaCl, 2 mM MgCl_2_, 5% DMSO or inhibitor, and 2 nM of purified nsp13, with or without 10 µg/mL BSA and 180 µM TCEP. The reaction was started by adding 200 µM ATP. After 30 min of incubation at 37 °C, 50 µL of Biomol^®^ Green Reagent (Prod. No. BML-AK111, Enzo Lifescience, Farmingdale, NY, USA) was added and the reaction was incubated for 10 min at RT, protected from the light. Products were measured with Victor Nivo (Perkin) at 650 nm for an ABS value. The experiments were conducted as N greater or equal to 3 biological independent replicates of three technical replicates for each tested condition.

#### 3.2.4. Data Analysis

Data analysis of the assay development results was performed using GraphPad Prism Version 9.1.2 (GraphPad Software, Inc., San Diego, CA, USA). Test compound results were normalized relative to the respective controls. Dose response curves were fitted to a nonlinear regression of (log_10_) dose vs. a normalized response-variable slope. Assay quality was assessed using the Z′-factor calculation with Z′ > 0.5 as the threshold for acceptance.

### 3.3. Molecular Modeling

For the evaluation of ATPase inhibition, the crystal structure of the SARS-CoV-2 helicase in a complex with the non-hydrolyzable ATP analog AMP-PNP (PDB ID: 7NN0 [60]) was selected. Although a structure of nsp13 complexed with ATP has recently been resolved (PDB ID: 9I53), it lacks the Mg^2+^ ion, which is critical for the catalytic mechanism and was included in our *in vitro* assays; since Mg^2+^ binding induces essential conformational changes according to the most accredited mechanistic models [60], the use of a magnesium-containing structure was considered fundamental. Regarding unwinding inhibition, a previously published 3D model of the SARS-CoV-2 nsp13 protein was adopted [50], generated via the SwissModel [61] using the SARS-CoV-1 helicase (PDB ID: 6JYT [62]) as a template. This model was further refined by incorporating the natural ligands ADP, ssRNA, and the Mg^2+^ ion, with coordinates derived through BLOSUM62-based alignment in UCSF Chimera [63] using PDB entry 2XZO [64]. Then, several tools implemented in the Schrödinger suite [65] were employed. All nsp13 3D structures were prepared using the Protein Preparation Wizard, which involved assigning bond orders, adding hydrogens, and generating physiological pH states using the EPIK tool, followed by an overall protein minimization with restrained heavy atoms and the removal of all water molecules. Docking calculations for compounds **13b**, **15a**, and **17d** were subsequently performed using the IFD tool implemented in Maestro. To prepare the ligands, the Schrödinger LigPrep tool was employed, specifically accounting for all predicted tautomeric forms of the DKA chain. Also, pKa calculations for the enolic forms of **15a** were conducted using Jaguar, yielding a value of 5.98; consequently, it was treated as enolate under physiological conditions. The docking grid boxes were centered on the RecA2 domain in accordance with previously reported DKA inhibitors, and all runs were carried out using the standard IFD protocol with default settings. Final binding complexes were selected based on the best-scoring poses and meticulous visual inspection, with all molecular renderings generated using PyMOL Version 2.4.2 (Schrödinger, LLC) [66].

## 4. Conclusions

The recent COVID-19 pandemic, caused by the SARS-CoV-2 virus, still poses a significant burden with devastating global consequences, prompting continuous efforts to identify new potential therapeutic tools for addressing the disease. Owing to its key role in the viral life cycle and a high interspecific similarity among coronaviruses, targeting the SARS-CoV-2 nsp13 enzyme has emerged as a relevant strategy for the search of innovative enzyme inhibitors which could potentially serve as therapeutic tools for contrasting SARS-CoV-2 infections.

Building upon the established efficacy of indole-based DKAs, we screened five series of pyrrolyl DKA and non-DKA derivatives against nsp13-associated activities. Through primary and secondary biochemical assays, we established preliminary SAR insights, identifying eight derivatives (**13a**,**b**, **15a**,**b**, **17b**–**d**,**g**) featuring inhibitory potency against both unwinding and ATPase functions, with IC_50_ values below 10 µM.

Although biochemical evaluation in the presence of BSA/TCEP revealed variations in potency among the derivatives, a generally consistent activity trend was observed across both assay conditions.

Interestingly, the compounds evaluated in the secondary assay showed inhibitory potencies within the same order of magnitude of the reference inhibitor SSYA10–001 against unwinding, with four derivatives (**13a**,**b**, **17c**,**d**) exhibiting statistically comparable activity to the reference compound. On the other hand, all derivatives evaluated in the secondary assay (**13a**–**c**, **15a**,**b**, **17b**–**d**,**g**) displayed statistically higher activity than licoflavone C against ATPase, displaying up to a 16-fold increase in potency compared to the reference inhibitor.

Despite the narrow potency range and the limited structural variations explored within this study, preliminary SAR indications were highlighted based on the IC_50_ values observed in the biochemical assays. Statistical analysis indicated that the most promising 2-DKE and 3-DKE derivatives (those with lower IC_50_ values) displayed no significant differences in their inhibitory potency against unwinding and ATPase. This suggests that alternative DKA derivatives could serve as potential scaffolds for inhibiting nsp13-associated activities in biochemical assays. In addition, preliminary observations indicated that compounds **13a**,**b**, **15a**,**b**, and **17b**–**d**,**g** showed similar IC_50_ values against both unwinding and ATPase, exhibiting favorable dual inhibitory profiles. This agrees with our previous findings about indolyl DKE derivatives, that proved to be promising dual inhibitors of both nsp13-associated activities. Moreover, these data confirm the usefulness of the scaffold hopping approach, suggesting the ring opening on indolyl DKA compounds is viable to achieve nsp13 inhibition.

Interestingly, no statistical difference was generally observed between the DKA derivatives and the investigated pyrazole analogs, preliminarily suggesting that replacing the DKA moiety with a pyrazole isostere could potentially represent a viable alternative strategy for inhibiting nsp13-associated activities. Complementing the preliminary SAR trends observed through biochemical assays, docking studies of representative derivatives from the 2-DKE, 3-DKE and pyrazole series suggested potential interactions within the RecA2 domain of nsp13, providing valuable insights for future medicinal chemistry efforts.

Overall, this study reports the biochemical evaluation of the inhibitory potency of pyrrolyl-containing DKA and non-DKA derivatives and outlines preliminary SAR observations. The promising inhibitory potency highlighted in two alternative biochemical assays, combined with the insights provided by docking studies, render these compounds preliminary biochemical hits featuring novel scaffolds distinct from those of previously reported inhibitors. Consequently, they serve as a promising starting point for developing novel, diversified antiviral tools to combat SARS-CoV-2. Based on these promising yet preliminary data, further investigations (*e.g*., assessments of the antiviral activity of the compounds in infected cells, cytotoxicity and selectivity profiling, mechanistic characterization, and target-engagement studies) will be useful to enhance their comprehensive characterization and investigate their potential application as antiviral tools.

## Figures and Tables

**Table 1:** Inhibition of SARS-CoV-2 nsp13 helicase-associated activities by pyrrolyl DKA and non-DKA derivatives **13a**–**f**, **14a**,**b**, **15a**–**c**, **16a**,**b**, and **17a**–**g**.

							IC_50_ (µM)			
**Cpd**	R	R_1_	R_2_	R_3_	R_4_	X	Unwinding ^a^	Unwinding BSA-TCEP ^b^	ATPase (%residual) ^c^	ATPase BSA-TCEP ^d^
**13a**	-	H	H	Cl	H	-	0.28 ± 0.19	3.10 ± 0.97	>30 (58%)	8.00 ± 3.6
**13b**	-	Cl	H	Cl	H	-	0.29 ± 0.19	3.51 ± 0.69	≥30 (51%)	6.90 ± 2.0
**13c**	-	Cl	H	H	H	-	0.70 ± 0.10	9.50 ± 2.8	>30 (84%)	21.90 ± 3.0
**13d**	-	Cl	H	H	Cl	-	2.19 ± 1.29	-	>30 (71%)	-
**13e**	-	H	H	CN	H	-	1.37 ± 0.50	-	>30 (78%)	-
**13f**	-	H	F	F	H	-	11.70 ± 1.0	-	>30 (95%)	-
**14a**	-	-	-	-	-	COOH	21.01 ± 6.4	-	>30 (96%)	-
**14b**	-	-	-	-	-	1*H*-1,2,4-triazol-3-yl	15.90 ± 1.5	-	>30 (96%)	-
**15a**	Ph	-	-	-	-	Et	1.42 ± 0.25	5.39 ± 1.01	3.39 ± 0.16 ^e^	1.84 ± 0.33
**15b**	Ph	-	-	-	-	H	0.98 ± 0.16	4.88 ± 0.19	≥30 (51%)	6.82 ± 1.16
**15c**	H	-	-	-	-	H	2.52 ± 1.2	-	>30 (86%)	-
**16a**	Ph	-	-	-	-	-	2.40 ± 0.59	-	>30 (88%)	-
**16b**	H	-	-	-	-	-	12.90 ± 2.8	-	>30 (81%)	-
**17a**	H	4-F-Ph	-	-	-	-	3.70 ± 1.9	-	>30 (100%)	-
**17b**	(CH_2_)_4_PhO	4-F-Ph	-	-	-	-	0.35 ± 0.042	8.60 ± 0.1	>30 (63%)	5.20 ± 1.3
**17c**	(CH_2_)_4_PhO	Napht-1-yl	-	-	-	-	0.60 ± 0.5	2.19 ± 0.49	>30 (68%)	3.60 ± 0.34
**17d**	(Naphthalen-1-yl)methoxy	Napht-1-yl	-	-	-	-	0.89 ± 0.12	1.15 ± 0.37	>30 (61%)	2.33 ± 0.66
**17e**	2-((4-carboxyphenyl)amino)-2-oxoethoxy	4-F-Ph	-	-	-	-	16.40 ± 3.6	-	>30 (82%)	-
**17f**	2-oxo-2-(phenylamino)ethoxy	4-F-Ph	-	-	-	-	2.13 ± 1.14	-	>30 (88%)	-
**17g**	2-oxo-2-(phenylamino)ethoxy	Napht-1-yl	-	-	-	-	2.10 ± 0.86	6.03 ± 1.32	>30 (59%)	4.98 ± 1.42
SSYA10–001 [37,50]	-	-	-	-	-	-	0.05 ± 0.02	1.73 ± 0.34	>3 (90%)	3.78 ± 1.5
Licoflavone C [37]	-	-	-	-	-	-	1.34 ± 0.31	9.9 ± 0.5	24.6 ± 3.8	29.0 ± 2.1

^a^ Compound concentration required to inhibit SARS-CoV-2 nsp13-associated unwinding activity by 50%. ^b^ Compound concentration required to inhibit SARS-CoV-2 nsp13-associated unwinding activity by 50% in the presence of 10 μg/mL of BSA and 180 μM TCEP. ^c^ Percentage of SARS-CoV-2 nsp13-associated ATPase residual activity measured at 30 µM. ^d^ Compound concentration required to inhibit SARS-CoV-2 nsp13-associated ATPase activity by 50% in the presence of 10 μg/mL of BSA and 180 μM TCEP. ^e^ Compound concentration required to inhibit SARS-CoV-2 nsp13-associated ATPase activity by 50%.

## Data Availability

Data are contained within the article.

## Supplementary Materials

The following supporting information can be downloaded at https://www.mdpi.com/article/10.3390/molecules31132376/s1: Figure S1: Dose response curves of active compounds tested in SARS-CoV-2 nsp13 unwinding assay in absence of BSA/TCEP; Table S1: Data analysis of assay development results was performed using GraphPad Prism Version 9.1.2; Figure S2: Dose response curves of active compounds tested in SARS-CoV-2 nsp13 ATPase assay in absence of BSA/TCEP; Table S2: Data analysis of assay development results was performed using GraphPad Prism Version 9.1.2; Figure S3: Dose response curves of active compounds tested in SARS-CoV-2 nsp13 unwinding assay in presence of BSA/TCEP; Table S3: Data analysis of assay development results was performed using GraphPad Prism Version 9.1.2; Figure S4: Dose response curves of active compounds tested in SARS-CoV-2 nsp13 ATPase assay in presence of BSA/TCEP; Table S4: Data analysis of assay development results was performed using GraphPad Prism Version 9.1.2. Table S5: One-way ANOVA and Tukey’s test for unwinding inhibition by compounds **13a**–**f**, **14a**,**b**, **15a**–**c**, **16a**,**b**, and **17a**–**g** in absence of BSA/TCEP. Data analysis was performed using GraphPad Prism Version 9.1.2; Table S6: One-way ANOVA and Tukey’s test for unwinding inhibition by compounds **13a**–**c**, **15a**,**b**, and **17b**–**d**,**g** in the presence of BSA/TCEP. Data analysis was performed using GraphPad Prism Version 9.1.2. Table S7: One-way ANOVA and Tukey’s test for ATPase inhibition by compounds **13a**–**c**, **15a**,**b**, and **17b**–**d**,**g** in the presence of BSA/TCEP. Data analysis was performed using GraphPad Prism Version 9.1.2.

## Abbreviations

The following abbreviations are used in this manuscript:

COVID-19	Coronavirus Disease 2019
DKA	Diketo acid
DKB	Diketobutanoic
DKE	Diketohexenoic
FDA	US Food and Drug Administration
M^pro^	Main protease
Nsp13	Non-structural protein 13
RdRp	RNA-dependent RNA polymerase
SAR	Structure–activity relationship
SARS-CoV-2	Severe acute respiratory syndrome coronavirus 2
