# Supplementary material for: Structure–Activity Relationships of Pyrrolyl-Containing Diketo Acid and Non-Diketo Acid Derivatives as Inhibitors of SARS-CoV-2 nsp13-Associated Activities
Source: Molecules. 2026 Jul 6;31(13):2376. doi: 10.3390/molecules31132376 (PMC13363528; doi:10.3390/molecules31132376)
Supplement: Supplementary file 1 [file molecules-31-02376-s001.zip › molecules-4384551-supplementary.pdf]

Article

Structure-activity relationships of pyrrolyl-containing diketoacid and non-diketoacid derivatives as inhibitors of SARS-CoV-2 nsp13-associated activities

Elisa Patacchini[1], Francesco Saccoliti[2,*], Roberta Emmolo[3], Valentina Noemi Madia[4], Emanuele Cara[1], Aurora Albano[1], Angela Corona[3], Enzo Tramontano[3], Roberto Di Santo[1], and Roberta Costi[1]

1 Dipartimento di Chimica e Tecnologie del Farmaco, Istituto Pasteur-Fondazione Cenci Bolognetti, "Sapienza" Università di Roma, p.le Aldo Moro 5, I-00185, Rome, Italy; elisa.patacchini@uniroma1.it; emanuele.cara@uniroma1.it; aurora.albano@uniroma1.it; roberto.disanto@uniroma1.it; roberta.costi@uniroma1.it

2 Department of Life Sciences, Health and Health Professions, Link Campus University, Via del Casale di San Pio V 44, I-00165, Rome, Italy; f.saccoliti@unilink.it

3 Laboratorio di Virologia Molecolare, Dipartimento di Scienze della Vita e dell'Ambiente Sezione biomedica, Università di Cagliari Cittadella Universitaria di Monserrato, Monserrato, Italy; roberta.emmolo@unica.it; angela.corona@unica.it; tramon@unica.it

4 Department of Science, Università degli Studi Roma Tre, Viale Guglielmo Marconi 446, I-00146, Rome, Italy; valentinanoemi.madia@uniroma3.it

* Correspondence: f.saccoliti@unilink.it

Supplementary Material:

Figure S1. Dose response curves of active compounds tested in SARS-CoV-2 nsp13 unwinding assay in absence of BSA/TCEP
Table S1. Data analysis of assay development results was performed using GraphPad Prism Version 9.1.2.
Figure S2. Dose response curves of active compounds tested in SARS-CoV-2 nsp13 ATPase assay in absence of BSA/TCEP
Table S2. Data analysis of assay development results was performed using GraphPad Prism Version 9.1.2.
Figure S3. Dose response curves of active compounds tested in SARS-CoV-2 nsp13 unwinding assay in presence of BSA/TCEP
Table S3. Data analysis of assay development results was performed using GraphPad Prism Version 9.1.2
Figure S4. Dose response curves of active compounds tested in SARS-CoV-2 nsp13 ATPase assay in presence of BSA/TCEP
Table S4. Data analysis of assay development results was performed using GraphPad Prism Version 9.1.2.
Table S5. One-way ANOVA and Tukey's test for unwinding inhibition by compounds **13a-f, 14a,b, 15a-c**, **16a,b**, and **17a-g** in absence of BSA/TCEP. Data analysis was performed using GraphPad Prism Version 9.1.2.
Table S6. One-way ANOVA and Tukey's test for unwinding inhibition by compounds **13a-c**, **15a,b**, **17b-d,g** in the presence of BSA/TCEP. Data analysis was performed using GraphPad Prism Version 9.1.2.
Table S7. One-way ANOVA and Tukey's test for ATPase inhibition by compounds **13a-c**, **15a,b**, **17b-d,g** in the presence of BSA/TCEP. Data analysis was performed using GraphPad Prism Version 9.1.2.

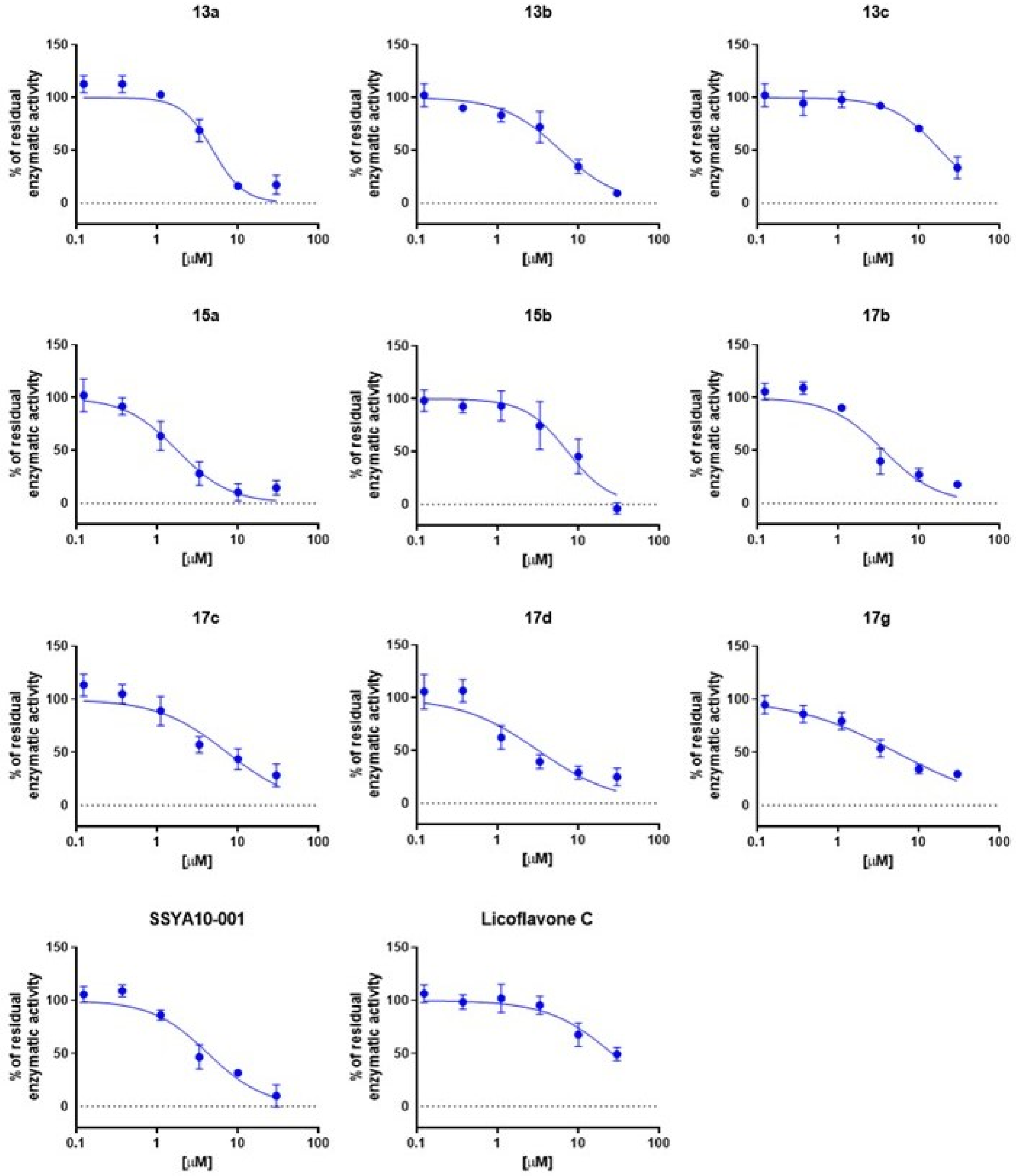

Figure S1. Dose response curves of active compounds tested in SARS-CoV-2 nsp13 unwinding assay in absence of BSA/TCEP. Dose response curves were fitted to a nonlinear regression of (log10)dose vs normalized response-variable slope.

Table S1. Data analysis of assay development results was performed using GraphPad Prism Version 9.1.2. Dose response curves were fitted to a nonlinear regression of (log10)dose vs normalized response-variable slope. Hill Slope, R squared and Confidence Intervals (CI %), extracted from the Table of results derived from the analysis, are reported in the following table.

Compound	Hill Slope	R squared	CI %
13a	-1,575	0,8965	98,958
13b	-1,292	0,9323	98,224
13c	-1,902	0,9337	98,083
13d	-1,292	0,8959	99,841
13e	-2,008	0,9393	98,556

13f	-2,510	0,7488	99,999
14a	-2,636	0,7066	99,999
14b	-1,802	0,7995	99,999
15a	-1,466	0,9155	99,999
15b	-4,918	0,9471	99,999
15c	-1,011	0,8317	99,476
16a	-3,520	0,9558	99,999
16b	-2,253	0,8195	99,999
17a	-1,413	0,8095	99,975
17b	-0,840	0,8942	94,260
17c	-1,997	0,9593	99,905
17d	-3,509	0,9627	99,999
17e	-2,765	0,9082	98,887
17f	-1,818	0,8804	99,987
17g	-2,365	0,9001	99,998
SSYA10−001	-2,028	0,9841	99,914
Licoflavone C	-1,386	0,9242	99,978

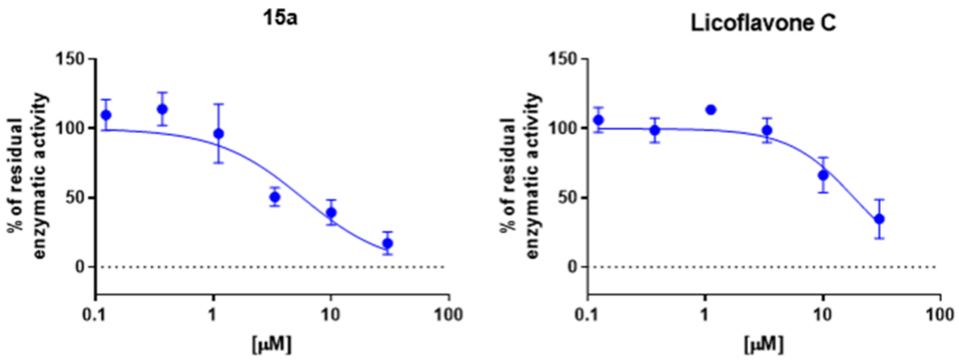

Figure S2. Dose response curves of active compounds tested in SARS-CoV-2 nsp13 ATPase assay in absence of BSA/TCEP. Dose response curves were fitted to a nonlinear regression of (log10)dose vs normalized response-variable slope.

Table S2. Data analysis of assay development results was performed using GraphPad Prism Version 9.1.2. Dose response curves were fitted to a nonlinear regression of (log10)dose vs normalized response-variable slope. Hill Slope, R squared and Confidence Intervals (CI %), extracted from the Table of results derived from the analysis, are reported in the following table.

Compound	Hill Slope	R squared	CI %
15a	-1,198	0,8331	98,083
Licoflavone C	-1,572	0,8685	99,841

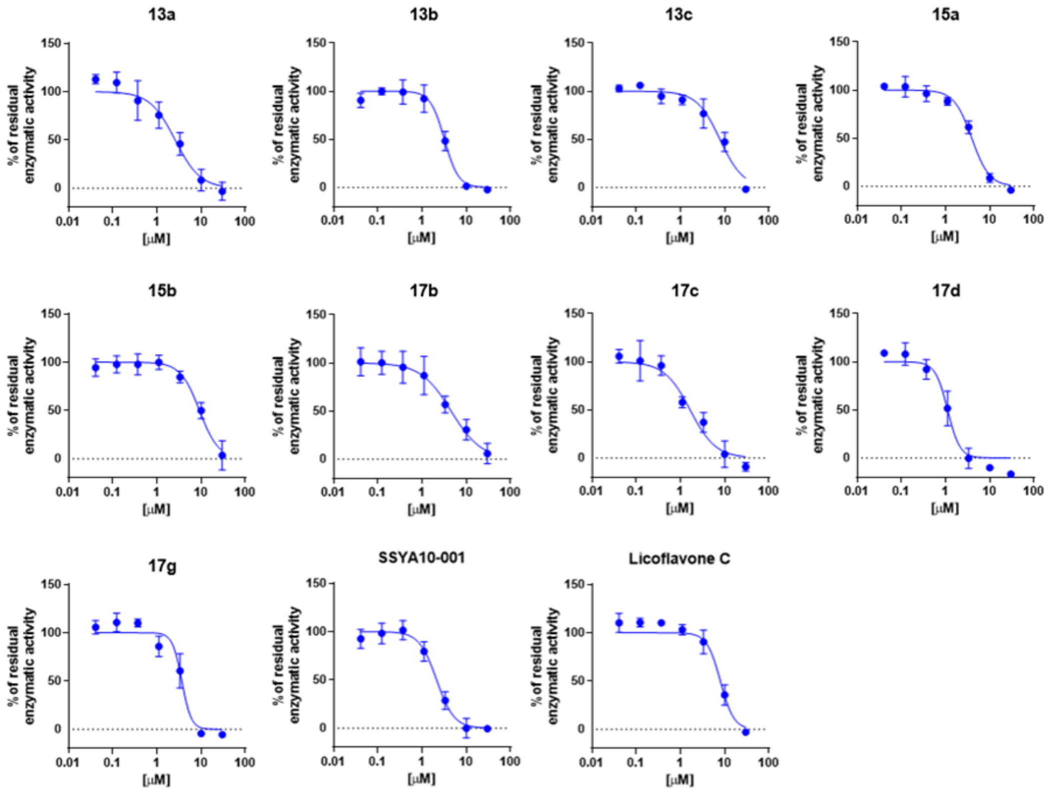

Figure S3. Dose response curves of active compounds tested in SARS-CoV-2 nsp13 unwinding assay in presence of BSA/TCEP. Dose response curves were fitted to a nonlinear regression of (log10)dose vs normalized response-variable slope.

Table S3. Data analysis of assay development results was performed using GraphPad Prism Version 9.1.2. Dose response curves were fitted to a nonlinear regression of (log10)dose vs normalized response-variable slope. Hill Slope, R squared and Confidence Intervals (CI %), extracted from the Table of results derived from the analysis, are reported in the following table.

Compound	Hill Slope	R squared	CI %
13a	-1,526	0,9159	99,966
13b	-2,744	0,9707	99,999
13c	-1,667	0,9451	99,997
15a	-2,173	0,9742	99,999
15b	-2,019	0,9294	99,999
17b	-1,270	0,8944	99,598
17c	-1,497	0,9339	99,923
17d	-3,092	0,9382	99,999
17g	-4,091	0,9441	99,999
SSYA10–001	-2,203	0,9629	99,998
Licoflavone C	-2,863	0,9522	99,999

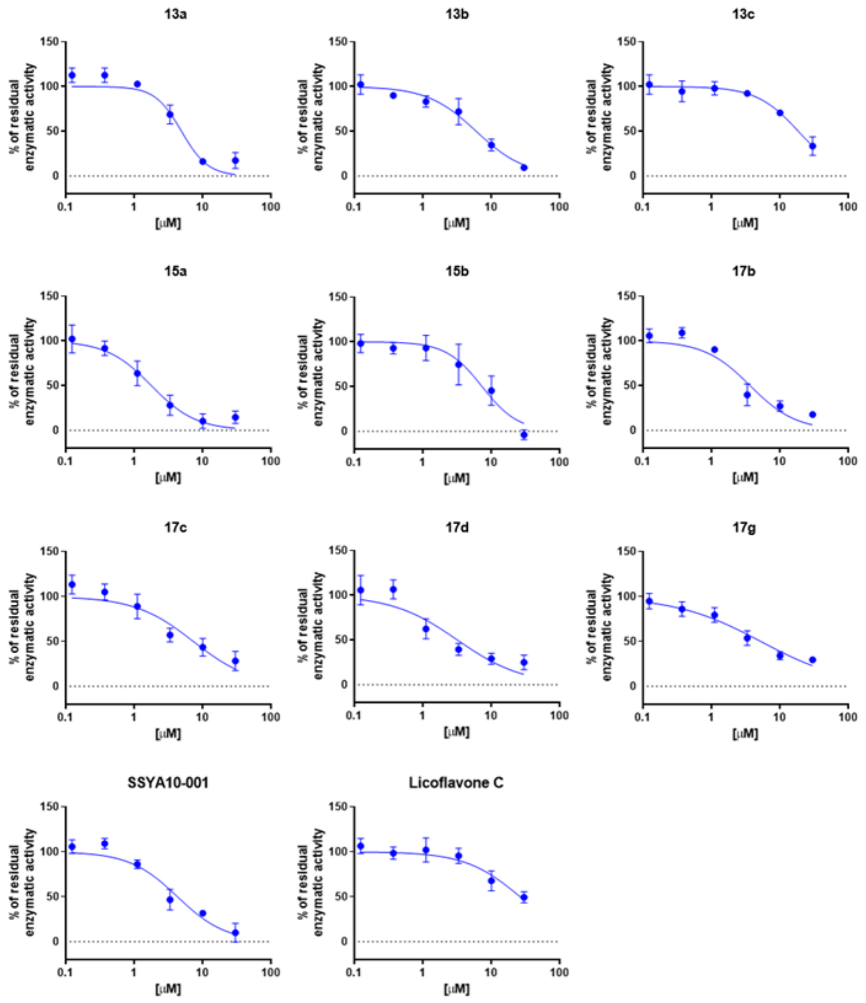

Figure S4. Dose response curves of active compounds tested in SARS-CoV-2 nsp13 ATPase assay in presence of BSA/TCEP. Dose response curves were fitted to a nonlinear regression of (log10)dose vs normalized response-variable slope.

Table S4. Data analysis of assay development results was performed using GraphPad Prism Version 9.1.2. Dose response curves were fitted to a nonlinear regression of (log10)dose vs normalized response-variable slope. Hill Slope, R squared and Confidence Intervals (CI %), extracted from the Table of results derived from the analysis, are reported in the following table.

Compound	Hill Slope	R squared	CI %
13a	-2,240	0,9199	99,874
13b	-1,219	0,9366	99,987
13c	-1,419	0,9359	99,888
15a	-1,328	0,9182	99,979
15b	-1,654	0,8592	99,986
17b	-1,296	0,8996	99,854
17c	-1,017	0,8334	99,999
17d	-0,906	0,8157	99,854
17g	-0,694	0,9185	98,568

SSYA10−001	-1,238	0,9319	98,085
Licoflavone C	-1,085	0,8237	99,244

Table S5. One-way ANOVA and Tukey's test for unwinding inhibition by compounds 13a-f, 14a,b, 15a-c, 16a,b, and 17a-g in absence of BSA/TCEP. Data analysis was performed using GraphPad Prism Version 9.1.2.

Tukey's multiple comparisons test	*Mean diff,*	*95,00% CI of diff,*	*Below threshold?*	*Summary*	*Adjusted P Value*
13a vs. 13b	-0,01	-5,769 to 5,749	No	ns	>0,9999
13a vs. 13c	-0,42	-6,179 to 5,339	No	ns	>0,9999
13a vs. 13d	-1,91	-7,669 to 3,849	No	ns	0,9994
13a vs. 13e	-1,09	-6,849 to 4,669	No	ns	>0,9999
13a vs. 13f	-11,42	-17,18 to -5,661	Yes	****	<0,0001
13a vs. 14a	-20,73	-26,49 to -14,97	Yes	****	<0,0001
13a vs. 14b	-15,62	-21,38 to -9,861	Yes	****	<0,0001
13a vs. 15a	-1,14	-6,899 to 4,619	No	ns	>0,9999
13a vs. 15b	-0,7	-6,459 to 5,059	No	ns	>0,9999
13a vs. 15c	-2,24	-7,999 to 3,519	No	ns	0,9955
13a vs. 16a	-2,12	-7,879 to 3,639	No	ns	0,9977
13a vs. 16b	-12,62	-18,38 to -6,861	Yes	****	<0,0001
13a vs. 17a	-3,42	-9,179 to 2,339	No	ns	0,7809
13a vs. 17b	-0,07	-5,829 to 5,689	No	ns	>0,9999
13a vs. 17c	-0,32	-6,079 to 5,439	No	ns	>0,9999
13a vs. 17d	-0,61	-6,369 to 5,149	No	ns	>0,9999
13a vs. 17e	-16,12	-21,88 to -10,36	Yes	****	<0,0001
13a vs. 17f	-1,85	-7,609 to 3,909	No	ns	0,9996
13a vs. 17g	-1,82	-7,579 to 3,939	No	ns	0,9997
13a vs. ssya10-001	0,23	-5,529 to 5,989	No	ns	>0,9999
13a vs. licoflavone C	-1,06	-6,819 to 4,699	No	ns	>0,9999
13b vs. 13c	-0,41	-6,169 to 5,349	No	ns	>0,9999
13b vs. 13d	-1,9	-7,659 to 3,859	No	ns	0,9995
13b vs. 13e	-1,08	-6,839 to 4,679	No	ns	>0,9999
13b vs. 13f	-11,41	-17,17 to -5,651	Yes	****	<0,0001
13b vs. 14a	-20,72	-26,48 to -14,96	Yes	****	<0,0001
13b vs. 14b	-15,61	-21,37 to -9,851	Yes	****	<0,0001
13b vs. 15a	-1,13	-6,889 to 4,629	No	ns	>0,9999
13b vs. 15b	-0,69	-6,449 to 5,069	No	ns	>0,9999
13b vs. 15c	-2,23	-7,989 to 3,529	No	ns	0,9957
13b vs. 16a	-2,11	-7,869 to 3,649	No	ns	0,9979
13b vs. 16b	-12,61	-18,37 to -6,851	Yes	****	<0,0001
13b vs. 17a	-3,41	-9,169 to 2,349	No	ns	0,7848
13b vs. 17b	-0,06	-5,819 to 5,699	No	ns	>0,9999
13b vs. 17c	-0,31	-6,069 to 5,449	No	ns	>0,9999
13b vs. 17d	-0,6	-6,359 to 5,159	No	ns	>0,9999
13b vs. 17e	-16,11	-21,87 to -10,35	Yes	****	<0,0001
13b vs. 17f	-1,84	-7,599 to 3,919	No	ns	0,9997
13b vs. 17g	-1,81	-7,569 to 3,949	No	ns	0,9997
13b vs. ssya10-001	0,24	-5,519 to 5,999	No	ns	>0,9999
13b vs. licoflavone C	-1,05	-6,809 to 4,709	No	ns	>0,9999
13c vs. 13d	-1,49	-7,249 to 4,269	No	ns	>0,9999
13c vs. 13e	-0,67	-6,429 to 5,089	No	ns	>0,9999
13c vs. 13f	-11	-16,76 to -5,241	Yes	****	<0,0001
13c vs. 14a	-20,31	-26,07 to -14,55	Yes	****	<0,0001
13c vs. 14b	-15,2	-20,96 to -9,441	Yes	****	<0,0001

13c vs. 15a	-0,72	-6,479 to 5,039	No	ns	>0,9999
13c vs. 15b	-0,28	-6,039 to 5,479	No	ns	>0,9999
13c vs. 15c	-1,82	-7,579 to 3,939	No	ns	0,9997
13c vs. 16a	-1,7	-7,459 to 4,059	No	ns	0,9999
13c vs. 16b	-12,2	-17,96 to -6,441	Yes	****	<0,0001
13c vs. 17a	-3	-8,759 to 2,759	No	ns	0,913
13c vs. 17b	0,35	-5,409 to 6,109	No	ns	>0,9999
13c vs. 17c	0,1	-5,659 to 5,859	No	ns	>0,9999
13c vs. 17d	-0,19	-5,949 to 5,569	No	ns	>0,9999
13c vs. 17e	-15,7	-21,46 to -9,941	Yes	****	<0,0001
13c vs. 17f	-1,43	-7,189 to 4,329	No	ns	>0,9999
13c vs. 17g	-1,4	-7,159 to 4,359	No	ns	>0,9999
13c vs. ssya10-001	0,65	-5,109 to 6,409	No	ns	>0,9999
13c vs. licoflavone C	-0,64	-6,399 to 5,119	No	ns	>0,9999
13d vs. 13e	0,82	-4,939 to 6,579	No	ns	>0,9999
13d vs. 13f	-9,51	-15,27 to -3,751	Yes	****	<0,0001
13d vs. 14a	-18,82	-24,58 to -13,06	Yes	****	<0,0001
13d vs. 14b	-13,71	-19,47 to -7,951	Yes	****	<0,0001
13d vs. 15a	0,77	-4,989 to 6,529	No	ns	>0,9999
13d vs. 15b	1,21	-4,549 to 6,969	No	ns	>0,9999
13d vs. 15c	-0,33	-6,089 to 5,429	No	ns	>0,9999
13d vs. 16a	-0,21	-5,969 to 5,549	No	ns	>0,9999
13d vs. 16b	-10,71	-16,47 to -4,951	Yes	****	<0,0001
13d vs. 17a	-1,51	-7,269 to 4,249	No	ns	>0,9999
13d vs. 17b	1,84	-3,919 to 7,599	No	ns	0,9997
13d vs. 17c	1,59	-4,169 to 7,349	No	ns	>0,9999
13d vs. 17d	1,3	-4,459 to 7,059	No	ns	>0,9999
13d vs. 17e	-14,21	-19,97 to -8,451	Yes	****	<0,0001
13d vs. 17f	0,06	-5,699 to 5,819	No	ns	>0,9999
13d vs. 17g	0,09	-5,669 to 5,849	No	ns	>0,9999
13d vs. ssya10-001	2,14	-3,619 to 7,899	No	ns	0,9974
13d vs. licoflavone C	0,85	-4,909 to 6,609	No	ns	>0,9999
13e vs. 13f	-10,33	-16,09 to -4,571	Yes	****	<0,0001
13e vs. 14a	-19,64	-25,40 to -13,88	Yes	****	<0,0001
13e vs. 14b	-14,53	-20,29 to -8,771	Yes	****	<0,0001
13e vs. 15a	-0,05	-5,809 to 5,709	No	ns	>0,9999
13e vs. 15b	0,39	-5,369 to 6,149	No	ns	>0,9999
13e vs. 15c	-1,15	-6,909 to 4,609	No	ns	>0,9999
13e vs. 16a	-1,03	-6,789 to 4,729	No	ns	>0,9999
13e vs. 16b	-11,53	-17,29 to -5,771	Yes	****	<0,0001
13e vs. 17a	-2,33	-8,089 to 3,429	No	ns	0,9928
13e vs. 17b	1,02	-4,739 to 6,779	No	ns	>0,9999
13e vs. 17c	0,77	-4,989 to 6,529	No	ns	>0,9999
13e vs. 17d	0,48	-5,279 to 6,239	No	ns	>0,9999
13e vs. 17e	-15,03	-20,79 to -9,271	Yes	****	<0,0001
13e vs. 17f	-0,76	-6,519 to 4,999	No	ns	>0,9999
13e vs. 17g	-0,73	-6,489 to 5,029	No	ns	>0,9999
13e vs. ssya10-001	1,32	-4,439 to 7,079	No	ns	>0,9999
13e vs. licoflavone C	0,03	-5,729 to 5,789	No	ns	>0,9999
13f vs. 14a	-9,31	-15,07 to -3,551	Yes	****	<0,0001

13f vs. 14b	-4,2	-9,959 to 1,559	No	ns	0,4353
13f vs. 15a	10,28	4,521 to 16,04	Yes	****	<0,0001
13f vs. 15b	10,72	4,961 to 16,48	Yes	****	<0,0001
13f vs. 15c	9,18	3,421 to 14,94	Yes	****	<0,0001
13f vs. 16a	9,3	3,541 to 15,06	Yes	****	<0,0001
13f vs. 16b	-1,2	-6,959 to 4,559	No	ns	>0,9999
13f vs. 17a	8	2,241 to 13,76	Yes	***	0,0006
13f vs. 17b	11,35	5,591 to 17,11	Yes	****	<0,0001
13f vs. 17c	11,1	5,341 to 16,86	Yes	****	<0,0001
13f vs. 17d	10,81	5,051 to 16,57	Yes	****	<0,0001
13f vs. 17e	-4,7	-10,46 to 1,059	No	ns	0,2464
13f vs. 17f	9,57	3,811 to 15,33	Yes	****	<0,0001
13f vs. 17g	9,6	3,841 to 15,36	Yes	****	<0,0001
13f vs. ssya10-001	11,65	5,891 to 17,41	Yes	****	<0,0001
13f vs. licoflavone C	10,36	4,601 to 16,12	Yes	****	<0,0001
14a vs. 14b	5,11	-0,6491 to 10,87	No	ns	0,1404
14a vs. 15a	19,59	13,83 to 25,35	Yes	****	<0,0001
14a vs. 15b	20,03	14,27 to 25,79	Yes	****	<0,0001
14a vs. 15c	18,49	12,73 to 24,25	Yes	****	<0,0001
14a vs. 16a	18,61	12,85 to 24,37	Yes	****	<0,0001
14a vs. 16b	8,11	2,351 to 13,87	Yes	***	0,0005
14a vs. 17a	17,31	11,55 to 23,07	Yes	****	<0,0001
14a vs. 17b	20,66	14,90 to 26,42	Yes	****	<0,0001
14a vs. 17c	20,41	14,65 to 26,17	Yes	****	<0,0001
14a vs. 17d	20,12	14,36 to 25,88	Yes	****	<0,0001
14a vs. 17e	4,61	-1,149 to 10,37	No	ns	0,2757
14a vs. 17f	18,88	13,12 to 24,64	Yes	****	<0,0001
14a vs. 17g	18,91	13,15 to 24,67	Yes	****	<0,0001
14a vs. ssya10-001	20,96	15,20 to 26,72	Yes	****	<0,0001
14a vs. licoflavone C	19,67	13,91 to 25,43	Yes	****	<0,0001
14b vs. 15a	14,48	8,721 to 20,24	Yes	****	<0,0001
14b vs. 15b	14,92	9,161 to 20,68	Yes	****	<0,0001
14b vs. 15c	13,38	7,621 to 19,14	Yes	****	<0,0001
14b vs. 16a	13,5	7,741 to 19,26	Yes	****	<0,0001
14b vs. 16b	3	-2,759 to 8,759	No	ns	0,913
14b vs. 17a	12,2	6,441 to 17,96	Yes	****	<0,0001
14b vs. 17b	15,55	9,791 to 21,31	Yes	****	<0,0001
14b vs. 17c	15,3	9,541 to 21,06	Yes	****	<0,0001
14b vs. 17d	15,01	9,251 to 20,77	Yes	****	<0,0001
14b vs. 17e	-0,5	-6,259 to 5,259	No	ns	>0,9999
14b vs. 17f	13,77	8,011 to 19,53	Yes	****	<0,0001
14b vs. 17g	13,8	8,041 to 19,56	Yes	****	<0,0001
14b vs. ssya10-001	15,85	10,09 to 21,61	Yes	****	<0,0001
14b vs. licoflavone C	14,56	8,801 to 20,32	Yes	****	<0,0001
15a vs. 15b	0,44	-5,319 to 6,199	No	ns	>0,9999
15a vs. 15c	-1,1	-6,859 to 4,659	No	ns	>0,9999
15a vs. 16a	-0,98	-6,739 to 4,779	No	ns	>0,9999
15a vs. 16b	-11,48	-17,24 to -5,721	Yes	****	<0,0001
15a vs. 17a	-2,28	-8,039 to 3,479	No	ns	0,9944
15a vs. 17b	1,07	-4,689 to 6,829	No	ns	>0,9999

15a vs. 17c	0,82	-4,939 to 6,579	No	ns	>0,9999
15a vs. 17d	0,53	-5,229 to 6,289	No	ns	>0,9999
15a vs. 17e	-14,98	-20,74 to -9,221	Yes	****	<0,0001
15a vs. 17f	-0,71	-6,469 to 5,049	No	ns	>0,9999
15a vs. 17g	-0,68	-6,439 to 5,079	No	ns	>0,9999
15a vs. ssya10-001	1,37	-4,389 to 7,129	No	ns	>0,9999
15a vs. licoflavone C	0,08	-5,679 to 5,839	No	ns	>0,9999
15b vs. 15c	-1,54	-7,299 to 4,219	No	ns	>0,9999
15b vs. 16a	-1,42	-7,179 to 4,339	No	ns	>0,9999
15b vs. 16b	-11,92	-17,68 to -6,161	Yes	****	<0,0001
15b vs. 17a	-2,72	-8,479 to 3,039	No	ns	0,9631
15b vs. 17b	0,63	-5,129 to 6,389	No	ns	>0,9999
15b vs. 17c	0,38	-5,379 to 6,139	No	ns	>0,9999
15b vs. 17d	0,09	-5,669 to 5,849	No	ns	>0,9999
15b vs. 17e	-15,42	-21,18 to -9,661	Yes	****	<0,0001
15b vs. 17f	-1,15	-6,909 to 4,609	No	ns	>0,9999
15b vs. 17g	-1,12	-6,879 to 4,639	No	ns	>0,9999
15b vs. ssya10-001	0,93	-4,829 to 6,689	No	ns	>0,9999
15b vs. licoflavone C	-0,36	-6,119 to 5,399	No	ns	>0,9999
15c vs. 16a	0,12	-5,639 to 5,879	No	ns	>0,9999
15c vs. 16b	-10,38	-16,14 to -4,621	Yes	****	<0,0001
15c vs. 17a	-1,18	-6,939 to 4,579	No	ns	>0,9999
15c vs. 17b	2,17	-3,589 to 7,929	No	ns	0,9969
15c vs. 17c	1,92	-3,839 to 7,679	No	ns	0,9994
15c vs. 17d	1,63	-4,129 to 7,389	No	ns	>0,9999
15c vs. 17e	-13,88	-19,64 to -8,121	Yes	****	<0,0001
15c vs. 17f	0,39	-5,369 to 6,149	No	ns	>0,9999
15c vs. 17g	0,42	-5,339 to 6,179	No	ns	>0,9999
15c vs. ssya10-001	2,47	-3,289 to 8,229	No	ns	0,9861
15c vs. licoflavone C	1,18	-4,579 to 6,939	No	ns	>0,9999
16a vs. 16b	-10,5	-16,26 to -4,741	Yes	****	<0,0001
16a vs. 17a	-1,3	-7,059 to 4,459	No	ns	>0,9999
16a vs. 17b	2,05	-3,709 to 7,809	No	ns	0,9985
16a vs. 17c	1,8	-3,959 to 7,559	No	ns	0,9998
16a vs. 17d	1,51	-4,249 to 7,269	No	ns	>0,9999
16a vs. 17e	-14	-19,76 to -8,241	Yes	****	<0,0001
16a vs. 17f	0,27	-5,489 to 6,029	No	ns	>0,9999
16a vs. 17g	0,3	-5,459 to 6,059	No	ns	>0,9999
16a vs. ssya10-001	2,35	-3,409 to 8,109	No	ns	0,992
16a vs. licoflavone C	1,06	-4,699 to 6,819	No	ns	>0,9999
16b vs. 17a	9,2	3,441 to 14,96	Yes	****	<0,0001
16b vs. 17b	12,55	6,791 to 18,31	Yes	****	<0,0001
16b vs. 17c	12,3	6,541 to 18,06	Yes	****	<0,0001
16b vs. 17d	12,01	6,251 to 17,77	Yes	****	<0,0001
16b vs. 17e	-3,5	-9,259 to 2,259	No	ns	0,7489
16b vs. 17f	10,77	5,011 to 16,53	Yes	****	<0,0001
16b vs. 17g	10,8	5,041 to 16,56	Yes	****	<0,0001
16b vs. ssya10-001	12,85	7,091 to 18,61	Yes	****	<0,0001
16b vs. licoflavone C	11,56	5,801 to 17,32	Yes	****	<0,0001
17a vs. 17b	3,35	-2,409 to 9,109	No	ns	0,8074

17a vs. 17c	3,1	-2,659 to 8,859	No	ns	0,8877
17a vs. 17d	2,81	-2,949 to 8,569	No	ns	0,9501
17a vs. 17e	-12,7	-18,46 to -6,941	Yes	****	<0,0001
17a vs. 17f	1,57	-4,189 to 7,329	No	ns	>0,9999
17a vs. 17g	1,6	-4,159 to 7,359	No	ns	>0,9999
17a vs. ssya10-001	3,65	-2,109 to 9,409	No	ns	0,6846
17a vs. licoflavone C	2,36	-3,399 to 8,119	No	ns	0,9916
17b vs. 17c	-0,25	-6,009 to 5,509	No	ns	>0,9999
17b vs. 17d	-0,54	-6,299 to 5,219	No	ns	>0,9999
17b vs. 17e	-16,05	-21,81 to -10,29	Yes	****	<0,0001
17b vs. 17f	-1,78	-7,539 to 3,979	No	ns	0,9998
17b vs. 17g	-1,75	-7,509 to 4,009	No	ns	0,9998
17b vs. ssya10-001	0,3	-5,459 to 6,059	No	ns	>0,9999
17b vs. licoflavone C	-0,99	-6,749 to 4,769	No	ns	>0,9999
17c vs. 17d	-0,29	-6,049 to 5,469	No	ns	>0,9999
17c vs. 17e	-15,8	-21,56 to -10,04	Yes	****	<0,0001
17c vs. 17f	-1,53	-7,289 to 4,229	No	ns	>0,9999
17c vs. 17g	-1,5	-7,259 to 4,259	No	ns	>0,9999
17c vs. ssya10-001	0,55	-5,209 to 6,309	No	ns	>0,9999
17c vs. licoflavone C	-0,74	-6,499 to 5,019	No	ns	>0,9999
17d vs. 17e	-15,51	-21,27 to -9,751	Yes	****	<0,0001
17d vs. 17f	-1,24	-6,999 to 4,519	No	ns	>0,9999
17d vs. 17g	-1,21	-6,969 to 4,549	No	ns	>0,9999
17d vs. ssya10-001	0,84	-4,919 to 6,599	No	ns	>0,9999
17d vs. licoflavone C	-0,45	-6,209 to 5,309	No	ns	>0,9999
17e vs. 17f	14,27	8,511 to 20,03	Yes	****	<0,0001
17e vs. 17g	14,3	8,541 to 20,06	Yes	****	<0,0001
17e vs. ssya10-001	16,35	10,59 to 22,11	Yes	****	<0,0001
17e vs. licoflavone C	15,06	9,301 to 20,82	Yes	****	<0,0001
17f vs. 17g	0,03	-5,729 to 5,789	No	ns	>0,9999
17f vs. ssya10-001	2,08	-3,679 to 7,839	No	ns	0,9982
17f vs. licoflavone C	0,79	-4,969 to 6,549	No	ns	>0,9999
17g vs. ssya10-001	2,05	-3,709 to 7,809	No	ns	0,9985
17g vs. licoflavone C	0,76	-4,999 to 6,519	No	ns	>0,9999
ssya10-001 vs. licoflavone C	-1,29	-7,049 to 4,469	No	ns	>0,9999

Table S6. One-way ANOVA and Tukey's test for unwinding inhibition by compounds 13a-c, 15a,b, 17b-d,g in the presence of BSA/TCEP. Data analysis was performed using GraphPad Prism Version 9.1.2.

Tukey's multiple comparisons test	*Mean diff,*	*95,00% CI of diff,*	*Below threshold?*	*Summary*	*Adjusted P Value*
13a vs. 13b	-0,41	-3,560 to 2,740	No	ns	>0,9999
13a vs. 13c	-6,4	-9,550 to -3,250	Yes	****	<0,0001
13a vs. 15a	-2,29	-5,440 to 0,8596	No	ns	0,3062
13a vs. 15b	-1,78	-4,930 to 1,370	No	ns	0,6384
13a vs. 17b	-5,5	-8,650 to -2,350	Yes	***	0,0001
13a vs. 17c	0,91	-2,240 to 4,060	No	ns	0,9917
13a vs. 17d	1,95	-1,200 to 5,100	No	ns	0,5189
13a vs. 17g	-2,93	-6,080 to 0,2196	No	ns	0,0833
13a vs. ssya10-001	1,37	-1,780 to 4,520	No	ns	0,8845
13a vs. licoflavone C	-6,8	-9,950 to -3,650	Yes	****	<0,0001
13b vs. 13c	-5,99	-9,140 to -2,840	Yes	****	<0,0001
13b vs. 15a	-1,88	-5,030 to 1,270	No	ns	0,5679
13b vs. 15b	-1,37	-4,520 to 1,780	No	ns	0,8845
13b vs. 17b	-5,09	-8,240 to -1,940	Yes	***	0,0003
13b vs. 17c	1,32	-1,830 to 4,470	No	ns	0,9054
13b vs. 17d	2,36	-0,7896 to 5,510	No	ns	0,2702
13b vs. 17g	-2,52	-5,670 to 0,6296	No	ns	0,1994
13b vs. ssya10-001	1,78	-1,370 to 4,930	No	ns	0,6384
13b vs. licoflavone C	-6,39	-9,540 to -3,240	Yes	****	<0,0001
13c vs. 15a	4,11	0,9604 to 7,260	Yes	**	0,0045
13c vs. 15b	4,62	1,470 to 7,770	Yes	**	0,0012
13c vs. 17b	0,9	-2,250 to 4,050	No	ns	0,9923
13c vs. 17c	7,31	4,160 to 10,46	Yes	****	<0,0001
13c vs. 17d	8,35	5,200 to 11,50	Yes	****	<0,0001
13c vs. 17g	3,47	0,3204 to 6,620	Yes	*	0,0229
13c vs. ssya10-001	7,77	4,620 to 10,92	Yes	****	<0,0001
13c vs. licoflavone C	-0,4	-3,550 to 2,750	No	ns	>0,9999
15a vs. 15b	0,51	-2,640 to 3,660	No	ns	>0,9999
15a vs. 17b	-3,21	-6,360 to -0,06040	Yes	*	0,0433
15a vs. 17c	3,2	0,05040 to 6,350	Yes	*	0,0443
15a vs. 17d	4,24	1,090 to 7,390	Yes	**	0,0032
15a vs. 17g	-0,64	-3,790 to 2,510	No	ns	0,9995
15a vs. ssya10-001	3,66	0,5104 to 6,810	Yes	*	0,0142
15a vs. licoflavone C	-4,51	-7,660 to -1,360	Yes	**	0,0016
15b vs. 17b	-3,72	-6,870 to -0,5704	Yes	*	0,0122
15b vs. 17c	2,69	-0,4596 to 5,840	No	ns	0,1409
15b vs. 17d	3,73	0,5804 to 6,880	Yes	*	0,0119
15b vs. 17g	-1,15	-4,300 to 2,000	No	ns	0,9581
15b vs. ssya10-001	3,15	0,0004002 to 6,300	Yes	*	0,05
15b vs. licoflavone C	-5,02	-8,170 to -1,870	Yes	***	0,0004
17b vs. 17c	6,41	3,260 to 9,560	Yes	****	<0,0001
17b vs. 17d	7,45	4,300 to 10,60	Yes	****	<0,0001
17b vs. 17g	2,57	-0,5796 to 5,720	No	ns	0,1805
17b vs. ssya10-001	6,87	3,720 to 10,02	Yes	****	<0,0001
17b vs. licoflavone C	-1,3	-4,450 to 1,850	No	ns	0,9131
17c vs. 17d	1,04	-2,110 to 4,190	No	ns	0,9784
17c vs. 17g	-3,84	-6,990 to -0,6904	Yes	**	0,009
17c vs. ssya10-001	0,46	-2,690 to 3,610	No	ns	>0,9999
17c vs. licoflavone C	-7,71	-10,86 to -4,560	Yes	****	<0,0001
17d vs. 17g	-4,88	-8,030 to -1,730	Yes	***	0,0006
17d vs. ssya10-001	-0,58	-3,730 to 2,570	No	ns	0,9998
17d vs. licoflavone C	-8,75	-11,90 to -5,600	Yes	****	<0,0001
17g vs. ssya10-001	4,3	1,150 to 7,450	Yes	**	0,0027
17g vs. licoflavone C	-3,87	-7,020 to -0,7204	Yes	**	0,0083
ssya10-001 vs. licoflavone C	-8,17	-11,32 to -5,020	Yes	****	<0,0001

Table S7. One-way ANOVA and Tukey's test for ATPase inhibition by compounds 13a-c, 15a,b, 17b-d,g in the presence of BSA/TCEP. Data analysis was performed using GraphPad Prism Version 9.1.2.

Tukey's multiple comparisons test	*Mean diff,*	*95,00% CI of diff,*	*Below threshold?*	*Summary*	*Adjusted P Value*
13a vs. 13b	1,1	-4,349 to 6,549	No	ns	0,9995
13a vs. 13c	-13,9	-19,35 to -8,451	Yes	****	<0,0001
13a vs. 15a	6,16	0,7113 to 11,61	Yes	*	0,0182
13a vs. 15b	1,18	-4,269 to 6,629	No	ns	0,9992
13a vs. 17b	2,8	-2,649 to 8,249	No	ns	0,7477
13a vs. 17c	4,4	-1,049 to 9,849	No	ns	0,1903
13a vs. 17d	5,67	0,2213 to 11,12	Yes	*	0,0368
13a vs. 17g	3,02	-2,429 to 8,469	No	ns	0,6623
13a vs. SSYA10-001	4,22	-1,229 to 9,669	No	ns	0,2331
13a vs. licoflavone C	-21	-26,45 to -15,55	Yes	****	<0,0001
13b vs. 13c	-15	-20,45 to -9,551	Yes	****	<0,0001
13b vs. 15a	5,06	-0,3887 to 10,51	No	ns	0,0842
13b vs. 15b	0,08	-5,369 to 5,529	No	ns	>0,9999
13b vs. 17b	1,7	-3,749 to 7,149	No	ns	0,9854
13b vs. 17c	3,3	-2,149 to 8,749	No	ns	0,5485
13b vs. 17d	4,57	-0,8787 to 10,02	No	ns	0,1559
13b vs. 17g	1,92	-3,529 to 7,369	No	ns	0,9666
13b vs. SSYA10-001	3,12	-2,329 to 8,569	No	ns	0,6219
13b vs. licoflavone C	-22,1	-27,55 to -16,65	Yes	****	<0,0001
13c vs. 15a	20,06	14,61 to 25,51	Yes	****	<0,0001
13c vs. 15b	15,08	9,631 to 20,53	Yes	****	<0,0001
13c vs. 17b	16,7	11,25 to 22,15	Yes	****	<0,0001
13c vs. 17c	18,3	12,85 to 23,75	Yes	****	<0,0001
13c vs. 17d	19,57	14,12 to 25,02	Yes	****	<0,0001
13c vs. 17g	16,92	11,47 to 22,37	Yes	****	<0,0001
13c vs. SSYA10-001	18,12	12,67 to 23,57	Yes	****	<0,0001
13c vs. licoflavone C	-7,1	-12,55 to -1,651	Yes	**	0,0045
15a vs. 15b	-4,98	-10,43 to 0,4687	No	ns	0,0935
15a vs. 17b	-3,36	-8,809 to 2,089	No	ns	0,5243
15a vs. 17c	-1,76	-7,209 to 3,689	No	ns	0,9814
15a vs. 17d	-0,49	-5,939 to 4,959	No	ns	>0,9999
15a vs. 17g	-3,14	-8,589 to 2,309	No	ns	0,6137
15a vs. SSYA10-001	-1,94	-7,389 to 3,509	No	ns	0,9643
15a vs. licoflavone C	-27,16	-32,61 to -21,71	Yes	****	<0,0001
15b vs. 17b	1,62	-3,829 to 7,069	No	ns	0,9897
15b vs. 17c	3,22	-2,229 to 8,669	No	ns	0,5811
15b vs. 17d	4,49	-0,9587 to 9,939	No	ns	0,1714
15b vs. 17g	1,84	-3,609 to 7,289	No	ns	0,9748
15b vs. SSYA10-001	3,04	-2,409 to 8,489	No	ns	0,6543
15b vs. licoflavone C	-22,18	-27,63 to -16,73	Yes	****	<0,0001
17b vs. 17c	1,6	-3,849 to 7,049	No	ns	0,9906
17b vs. 17d	2,87	-2,579 to 8,319	No	ns	0,7212
17b vs. 17g	0,22	-5,229 to 5,669	No	ns	>0,9999
17b vs. SSYA10-001	1,42	-4,029 to 6,869	No	ns	0,9962
17b vs. licoflavone C	-23,8	-29,25 to -18,35	Yes	****	<0,0001
17c vs. 17d	1,27	-4,179 to 6,719	No	ns	0,9985
17c vs. 17g	-1,38	-6,829 to 4,069	No	ns	0,997
17c vs. SSYA10-001	-0,18	-5,629 to 5,269	No	ns	>0,9999
17c vs. licoflavone C	-25,4	-30,85 to -19,95	Yes	****	<0,0001
17d vs. 17g	-2,65	-8,099 to 2,799	No	ns	0,801
17d vs. SSYA10-001	-1,45	-6,899 to 3,999	No	ns	0,9956
17d vs. licoflavone C	-26,67	-32,12 to -21,22	Yes	****	<0,0001
17g vs. SSYA10-001	1,2	-4,249 to 6,649	No	ns	0,999
17g vs. licoflavone C	-24,02	-29,47 to -18,57	Yes	****	<0,0001
SSYA10-001 vs. licoflavone C	-25,22	-30,67 to -19,77	Yes	****	<0,0001
